# Machine-learned global glacier ice volumes

**DOI:** 10.1038/s41597-026-07744-9

**Published:** 2026-07-30

**Authors:** Niccolò Maffezzoli, Eric Rignot, Carlo Barbante, Mathieu Morlighem, Troels C. Petersen, Sebastiano Vascon

**Affiliations:** 1 Department of Environmental Sciences, Informatics and Statistics, Ca’ Foscari University of Venice, Via Torino 155, Venezia, 30172 Italy; 2 Institute of Polar Sciences, National Research Council, Venezia, Italy; 3 Department of Earth System Science, University of California Irvine, Croul Hall, Irvine, 92627 California USA; 4 Jet Propulsion Laboratory, Pasadena, USA; 5 Department of Civil and Environmental Engineering, University of California Irvine, Croul Hall, 92627 Irvine, USA; 6 Institut des Géosciences de l’Environnement, Saint-Martin-D’Heres, France; 7 Department of Earth and Planetary Sciences, Dartmouth College, Hanover, 03755 NH USA; 8 Niels Bohr Institute, University of Copenhagen, Copenhagen, Denmark

## Abstract

We present a global dataset of glacier ice thickness modeled with IceBoost v2.0, a gradient-boosted decision tree scheme trained on 7 million ice thickness measurements and informed by physical and geometrical predictors. We model the distributed ice thickness for every glacier in the two latest Randolph Glacier Inventory releases (RGI v6.0 and v7.0), totaling 215,547 and 274,531 glacier outlines, respectively, plus 955 ice masses contiguous with the Greenland Ice Sheet. IceBoost v2.0 represents the third existing RGI v6.0 global ice volume estimate, and the first for RGI v7.0. On RGI v6.0 we find a global glacier volume of (150 ± 38) × 10^3^ km^3^, consistent with the two previous estimates of (141 ± 40) × 10^3^ km^3^ and (158 ± 41) × 10^3^ km^3^. The corresponding sea-level equivalent (SLE), 323 ± 91 mm, is likewise consistent with the two earlier values of 311 ± 100 mm and 324 ± 84 mm. On RGI v7.0 we find a global glacier volume of (149 ± 38) × 10^3^ km^3^ and SLE of 323 ± 91 mm. Reconstructed ice thickness distributions can vary substantially across models for individual glaciers, ice caps, and even large glacier complexes. Compared to measurements, IceBoost v2.0 root mean square error is 20–45% lower than that of other models in the high Arctic, and comparable elsewhere. We examine major glaciated regions and compare results with the other models. Confidence in our estimates is highest at high latitudes, where abundant training data adequately sample the feature space. Over steep and mountainous terrain, small glaciers, and lower-latitude regions with limited training data, confidence is lower. IceBoost v2.0 is applicable to ice sheet margins. On the Geikie Plateau (East Greenland), we find nearly twice as much ice as previously reported, highlighting the potential for improved constraints on bed topography in this region. No physical laws are explicitly imposed during training, so sufficient and high-quality training data are crucial. The quality of the generated maps depends on the accuracy of the training data, the Digital Elevation Model, ice velocity fields, and glacier geometries, including nunataks. Using the Jensen Gap, we probe the model’s curvature with respect to input errors and find it is strongly concave over low-slope, thick-ice regions, implying a potential downward bias in predicted thickness under input uncertainty. The released dataset can be used to model future glacier evolution and sea-level rise, inform the design of glaciological surveys and field campaigns, as well as guide policies on freshwater management.

## Background & Summary

Knowledge of the volumes of glaciers and ice caps, as well as their spatially distributed ice thickness, is fundamental for geophysical modeling. Models projecting the future evolution of ice masses must be initialized with and are particularly sensitive to accurate present-day conditions^3^. This requirement is becoming progressively difficult to satisfy, as accelerated climate warming and glacier shrinkage cause present-day glacier states to evolve faster than our ability to produce updated thickness maps, many of which still reflect conditions from the early 2000s. Glaciers are retreating worldwide, having lost about 5% of their total mass over the past two decades and up to 39% in some regions of the world^4^. They account for approximately 25–30% of modern sea-level rise^5,6^. The rate of ice loss has accelerated during the last decade^4,7^, and projections indicate that glaciers may lose 26–41% of their total mass by 2100, depending on the undertaken future climate trajectory^8^. The implications are far-reaching, affecting freshwater availability and management^9–11^, coastal habitability^12^, and a wide range of socio-economic systems dependent on glacier-fed environments.

Inferring the global distribution of glacier ice thickness remains a major challenge. Only a few global-scale modeling efforts exist to date^1,2,13^, each relying on geometrical relationships, mathematical interpolations, physics-based models or mass conservation principles.

Over recent decades, millions of in-situ and airborne ice thickness measurements have been collected by international efforts such as the World Glacier Monitoring Service (WGMS^14^), NASA’s Operation IceBridge, and numerous regional and individual glaciological surveys. The GlaThiDa (Glacier Thickness Database) Consortium^15^ has consolidated most of these measurements into a unified dataset (currently v3.1.0, with v.4 forthcoming).

**Fig. 1:**
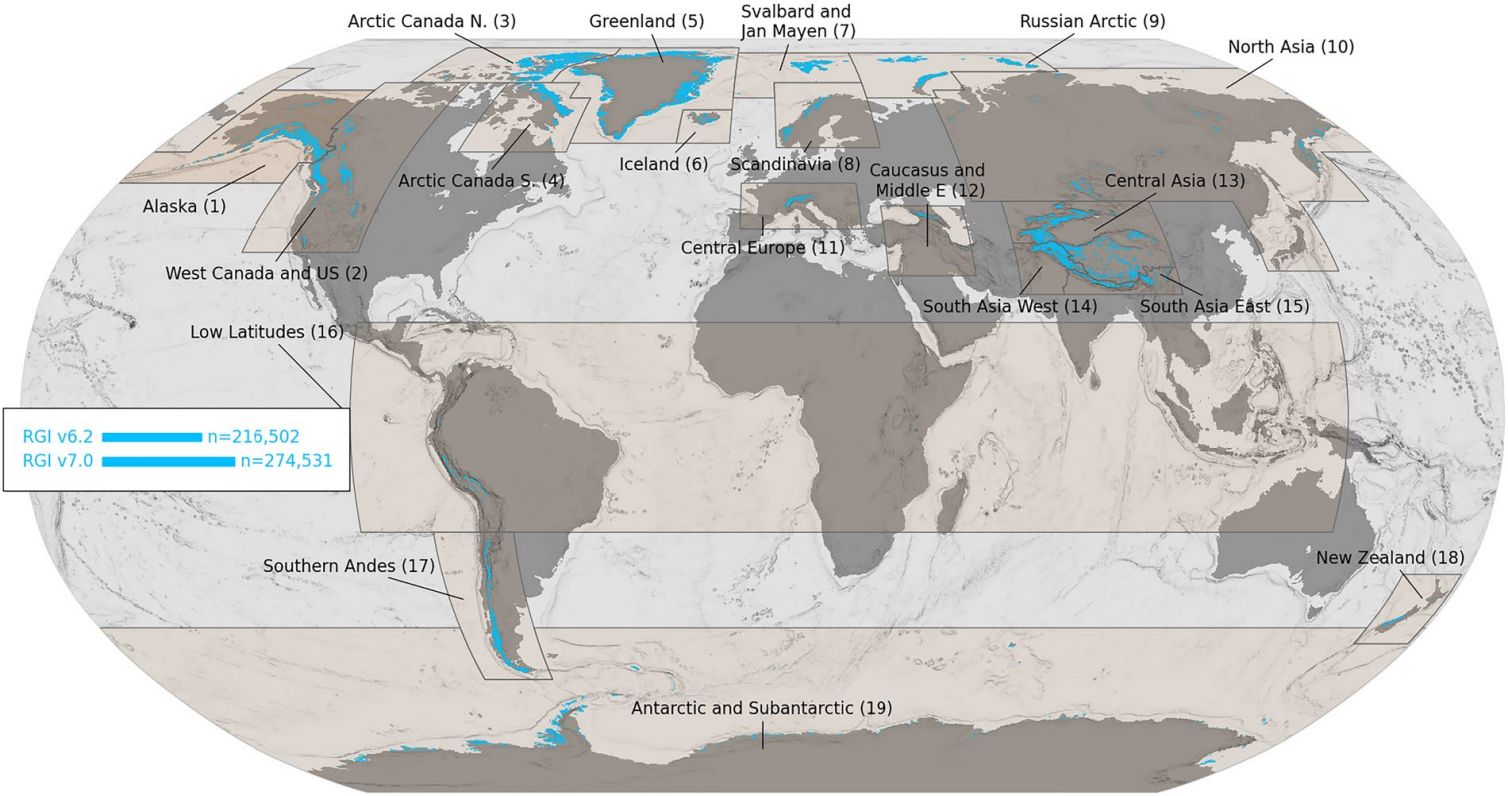
The World’s glaciers (cyan), divided into the 19 regions of the Randolph Glacier Inventory. The box indicates the total number of glaciers in versions v.6.2 and v.7.

In this study, we provide a new estimate of the distributed ice thickness of the world’s glaciers, using IceBoost v2.0 a machine learning model. We combine ice thickness observations from GlaThiDa and additional surveys, encompassing 1,661 glaciers worldwide and over seven million measurements, to build a training dataset and train a system of two gradient-boosted decision tree schemes. We then use this system to generate distributed ice thickness map for all glaciers globally^16^, as well as regional mosaics^17^. The model, IceBoost v2.0, represents an updated version that prioritizes smoothness of the predicted thickness field, over the previous version v1.1^18^. Alongside the global ice thickness maps derived for all glaciers in the Randolph Glacier Inventory (RGI, Fig. 1) version 6 (hereafter RGI v.6, *n* = 216,502) and version 7.0 (hereafter RGI v.7, *n* = 274,531), we provide maps of ice thickness uncertainty, surface elevation, geoid elevation, and Jensen Gap, a metric of model nonlinearity. We compare the IceBoost v2.0 estimate with the shallow ice approximation approach of Millan *et al*.^1^, and with the model ensemble of Farinotti *et al*.^2^, which includes the mass-conservation-based inversion of Huss *et al*.^13^ (Supplementary Information).

## Methods

### Training data

IceBoost v2.0 is trained with publicly available ice thickness datasets (Table 1). GlaThiDa v3.1.0 (n = 3,854,279 measurements^14^) represents the main global dataset. We also incorporate the following regional datasets: in Alaska, the surveys carried out on the Ruth glacier^19^ and other glaciers by^20^, totaling n = 1,472,965 extra measurements. In Scandinavia, we add the survey on the Jostedalsbreen ice cap (n = 351,559^21^). Over the two Patagonian ice fields, we add two datasets^22,23^ for a total of n = 418,689 extra measurements. At high latitudes we incorporate two main datasets. We use the IceBridge product^24^, which intercepts glaciers over the Canadian Arctic, coastal Greenland, Svalbard, and the Antarctic periphery. Furthermore, we add the 2002-2023 product that covers glaciers over the Antarctic peninsula acquired by the Center for Remote Sensing of Ice Sheets (CReSIS^25^). The Antarctic peninsula is not part of the official Randolph Glacier Inventory, but have a large number of alpine glaciers with a great number of measurements that offer the opportunity to extend the training dataset and train a model capable of generalizing to the ice sheet periphery. We note that in coastal Greenland, as part of IceBridge, we use multiple surveys taken on the Geikie Plateau, with direct connection to the Greenland ice sheet. Altogether, we train the model with n = 2,868,276 additional measurements located at very high latitudes (IceBridge and CReSIS), many of which located in regions proximal to the ice sheets. Globally, IceBoost v2.0 is trained on nearly twice as much data as IceBoost v1.1.

**Table 1:** Ice thickness datasets used as training targets for IceBoost v2.0.

Dataset	No. points	Domain	Data source	Reference
GlaThiDa v3.1.0	3,854,279	Global	GTN-G	^15^
Alaska	1,472,965	Alaska and Northwestern Canada	University Arizona Data repositoryIceBridge, IceBridge	^19,20,47,48^
Patagonia	418,689	Patagonian icefields	QFuego-Patagonia, UC Irvine Dryad Data Repository	^22,49^
Scandinavia	351,559	Jostedalsbreen ice cap	Norwegian Nasjonalt Vitenarkiv	^21^
Polar	2,868,276	Greenland, Canadian Arctic, Svalbard, Antarctica	IceBridge, CReSIS	^24,25^

Total points before cleaning 8,965,768.

Total points after cleaning 7,069,690 (n = 1,661 glaciers).

Total points after encoding 378,373 (n = 1,661 glaciers).

### Training data quality control and pre-processing

IceBoost v2.0 adopts a series of filters to maximize the quality of the training set. Data time-tagged older than 2005 is removed; data registered in GlaThiDa with less than 1 meter thickness is removed; data registered outside glacier polygons is removed (unless those collected over the Antarctic peninsula); data flagged as either erroneous or limited to parts of the glacier in the different datasets are completely removed. The final combined dataset was manually checked for outliers, for every individual glacier. The consistency of crossover tracks, if present, was used as a criterion. If crossover tracks were not present but close measurements were obtained, proximal measurements were judged for consistency. Some tracks were removed for inconsistencies between tracks, some measurements featured unreasonable data. Over the Antarctic peninsula, the echograms were checked and the questionable bed picks where manually removed, using the filtered dataset from BedMachine Antarctica^26^. Generally, data removal was aggressive: if data were found to be suspicious, they were removed. Most of the removed data were in the periphery of Greenland (along the southern and eastern coasts) and Antarctica, as well as in the Antarctic peninsula and over the two Patagonian icefields. Finally, measurements that did not have the complete set of valid inputs (e.g. ice velocity not available because of incomplete coverage) were also removed from the training set. Missing ice velocity was the first cause for deleting otherwise valid ground truth data. We acknowledge that despite the implemented quality control pipeline, some outliers can still be present in the final dataset.

As in IceBoost v1.1, IceBoost v2.0, encodes the training dataset by averaging both ice thickness data and the whole input feature vector in a 100 × 100 per-glacier pixel grid. This procedure is implemented to compensate for the different spatial resolution at which different ground penetrating radar devices acquire the thickness data along the route, that would otherwise result in a training dataset with less entries for less resolved tracks. The downscaling pipeline reduces the training dataset from 7,069,690 data points to its final size of 378,373 data points (Table 1, Supp. Info. Table S4), collected over 1,661 glaciers (less than 1% of all existing glaciers).

### Model inputs and training

IceBoost v2.0 was trained using 26 input variables (Table 2). For training, the variables were calculated and stored offline in a training dataset. At the inference time, all features were calculated on the fly. Elevation, slope, and curvature were derived from the freely available TanDEM-X 30m Edited DEM (EDEM) v1^27–29^, an automatically edited (filtered, interpolated, and infilled) variant of the global digital elevation model acquired between 2010 and 2014 under the TanDEM-X mission. As in IceBoost v1.1, we included multiple slope and curvature variables obtained by applying Gaussian kernels of varying sizes to the DEM. Smaller kernels capture fine-scale features, which are important for small glaciers, while larger kernels capture broader-scale variations relevant for extensive ice masses.

**Table 2:** IceBoost v2.0 model inputs, their units, resolution and referenced time. tag.

Feature	Variable	Unit	Resolution	Uncertainty	Time tag	Source
- Curvature	*c*_50_, *c*_100_, *c*_150_, *c*_300_, *c*_450_, c_gfa_	m^−1^	30 m	$2\sqrt{5}{\sigma }_{z}/\Delta {x}^{2}$ (Eq. 11)	2010–2014	Tandem-X Edited DEM^27–29^
- Distance to glacier margins/ or nunataks	*d*_*noice*_	km	30 m	100 m	2000–2010	RGI polygons v7.0, v6.0^30,33^
- Distance from ocean	*d*_*ocean*_	km	50–500 m	100 m	>2000	GSHHG^34^
- Surface Elevation	*z*	m	30 m	2–4 m (Eq. 9)	2010–2014	Tandem-X Edited DEM^27–29^
- Length	*lmax*	m	30 m	5%	2000–2010	RGI polygons v7.0, v6.0^30,33^
- Surface slopes	*s*_50_, *s*_75_, *s*_100_, *s*_125_, *s*_150_, *s*_300_, *s*_450_, *s*_*gfa*_	1	30 m	$2\sqrt{2}/\Delta x$ Eq. (10)	2010–2014	Tandem-X Edited DEM^27–29^
- Surface Mass balance	*smb*	kg/(m^2^ ⋅ yr)	30–2000 m	10%	1961–2021 2000–2019	RACMO2.3p2^37,38^ Hugonnet *et al*.^7^
- Temperature at 2 meters	*t*2*m*	K	9 km	1 K	2000–2010	ERA5^40^, ERA5-Land^39^
- Velocity	*v*_50_, *v*_100_, *v*_150_, *v*_300_, *v*_450_, *v*_*gfa*_	m/yr	50 m 250 m 450 m	10 m/yr 18 m/yr 18 m/yr	2017–2018	Millan *et al*.^1^ Joughin *et al*.^41^ Mouginot *et al*.^42^

The distance to glacier margins or internal rock outcrops (denoted *d*_*noice*_) was calculated using glacier polygons from the Randolph Glacier Inventory, v6.0^30,31^ and the most recent v7.0^32,33^. The distance to the ocean was derived from the Global Self-consistent Hierarchical High-resolution Geography (GSHHG) Shorelines product, v2.3.7, used at full (’f’) resolution^34^. These shorelines are based on and updated from the World Vector Shoreline project^35^.

We used the same distributed surface mass balance products as in IceBoost v1.1^18^. For glaciers at the peripheries of Greenland and Antarctica, we used RACMO2.3p2^36^, downscaled to 1 km^37^ and 2 km^38^, and averaged over 1961–1990 and 1979–2021, respectively. Outside the ice sheets, we fitted an empirical linear mass balance-elevation lapse rate for each glacier pair in the 19 regions. This involves determining the mass balance at zero elevation and the slope ($\frac{dMB}{dz}$) of the lapse rate using glacier-integrated geodetic mass balance data from Hugonnet *et al*.^7^, along with elevations from TanDEM-X EDEM. Within the same region, we imposed an inverse squared distance weight to encourage similar parameters for nearby glacier pairs and reduce the influence of distant glaciers.

Temperature inputs (2-m air temperature, *t2m*) were obtained from ERA5-Land (0. 1° grid spacing, ≈ 9 km^39^). For pixels missing due to imperfect land masks along coastlines and islands, we supplemented with the ERA5 t2m field (0.25° resolution^40^), bilinearly interpolated to the ERA5-Land 0. 1° grid.

Ice velocity inputs are identical to those used in IceBoost v1.1. Specifically, for glaciers in the Greenland periphery and Antarctica (both peripheral and continental), we used the products of Joughin *et al*.^41^, and Mouginot *et al*.^42^. For all other glaciers we used the product of Millan *et al*.^1^.

IceBoost v2.0 comprises independent XGBoost and CatBoost modules, both trained using a squared-loss objective within a 100-iteration cross-validation pipeline on a random 20% global subset of the training dataset. Model performance (deviation from ground truth data) was quantified regionally (Supp. Information, Sect. S2) by computing the root mean square error on 20% of regional measurements, using glacier-level separation between training and test sets and repeating the random splits 100 times. Hyperparameters are reported in the Supplementary Information, Tables S1–S2. At inference time, when the model is tasked to predict the ice thickness on a regular grid, the predictions from the two modules are equally averaged. The model was evaluated against the ground-truth gridded dataset, where available, and against alternative models, at the locations of the measurements (Supplementary Information, Section S5).

### Model updates from IceBoost v1.1 to v2.0

IceBoost v2.0 was refined to prioritize the smoothness of the ice thickness estimate across neighboring glaciers. In IceBoost v1.1, we observed that several input variables introduced discontinuities at glacier boundaries. We removed variables that were prone to DEM artifacts: *z*_*min*_ (minimum elevation), *z*_*max*_ (maximum elevation), *z* − *z*_*min*_ (elevation above glacier base), *z*_01_ (normalized elevation), and *Δz* (glacier elevation range). Additional features were excluded due to their sensitivity to imputation (glacier-integrated mass balance values), or the subjective nature of glacier delineations (glacier and cluster areas, *A*, *A*_*cluster*_, as well as the perimeter). Furthermore, variables found to have limited predictive importance^18^ based on SHAP (Shapley Additive Explanations) analysis but added to the computational burden were removed: glacier-integrated values of aspect, curvature, and slope.

The velocity product used for continental glaciers^1^ often contains partially or completely missing data, as well as very low values (<5 m/yr). To address this, we excluded the velocity features when data are missing or when the glacier-wide average velocity fell below 5 m/yr. This approach also removes the need for velocity imputation, which in IceBoost v1.1 was performed using regional averages. Moreover, to enrich the feature set for very small glaciers or clusters (<10 *km*^2^) lacking velocity information, we added the glacier length (*l*_*max*_, otherwise unused) as an additional predictor.

Although some information is discarded in the new model, IceBoost v2.0 ensures continuity of all input features across neighboring glaciers and glacier clusters, removing dependence and arbitrariness of individual glacier delineations and resulting in smooth transitions in the generated ice thickness maps. Any remaining discontinuities arise from other factors, such as the sharp decision boundaries inherent to decision-tree algorithms. Tested against ground truth data, IceBoost v2.0 root mean square error was similar to Millan and Farinotti’s models in non-polar regions, and up to 20–45% lower at high latitudes (Supplementary Information S2).

### Projection, posting and time tag

#### Study domain

We modeled the distributed ice thickness for all n=216,502 glaciers in RGI v6.0 and n=274,531 glaciers in RGI v7.0. In RGI v6.0, we retained 955 polygons located at the periphery of Greenland (notably the Geikie Plateau) and Antarctica, corresponding to connectivity level 2 ice-sheet marginal regions (see the RGI v6.0 user guide for a description of connectivity levels^30^). These regions provide valuable training data and enable training the model in the outer ice-sheet environment. These outlines are not part of version v7.0, which excludes all polygons with direct connection to the ice sheets. RGI v7.0 consists of 73% new or updated glacier outlines compared to version v6.0, equivalent to a 42 % improvement of glacier surface area globally. We also included training data in the Antarctic peninsula. We created n=50 geometries inside the peninsula by simply partitioning the area into randomly-sized Voronoi polygons. The peninsula outer geometry and inner rock outcrops (nunataks) were taken from BedMachine Antarctica v4^43,44^, truncated at northings=300,000 m (≃73–75^∘^S).

#### Updated glacier geometries from RGI v6.0 to RGI v7.0

The revised glacier geometries in RGI v7.0 can have a significant impact on the modeled ice thickness. Two main effects can be identified: updated glacier outlines and modifications of internal nunataks. Regarding the first effect, erroneously mapped glaciers in RGI v6.0 are typically modeled as too thick, as georeferencing errors can include flat, non-glaciated terrain (low slope values tend to increase the predicted thickness). Regarding the second effect, the increased number of internal nunataks in RGI v7.0 generally reduces the IceBoost v2.0 predicted ice thickness in surrounding areas, sometimes substantially. This is evident in the South Patagonia Icefield (e.g., Pío XI glacier), where v7.0 yields markedly thinner ice than v6.0, consistent with the increased representation of nunataks. The quality of the glacier geometry dataset is extremely important. For ice volume estimates, the role of nunataks geometries is larger than any revision of the outside glacier borders, which is marginal, as typically ice at the margins is shallow. A last effect regarding glacier geometries relates to misplaced geometries, not centered on glaciers. An example for this can be seen over glaciers on the Coronation Islands (Subantarctic Islands, 60.6°S 45.6° W), for which all RGI v6.0 geometries are shifted with respect to the real glacier positions, corrected in RGI v7.0. As a result, the distributed ice thickness predicted for erroneous geometries in RGI v6.0 should not be trusted. We generally observed a very significant improvement in the quality of glacier geometries in the latest RGI v7.0 dataset.

#### Time tag and time uncertainty

**Fig. 2:**
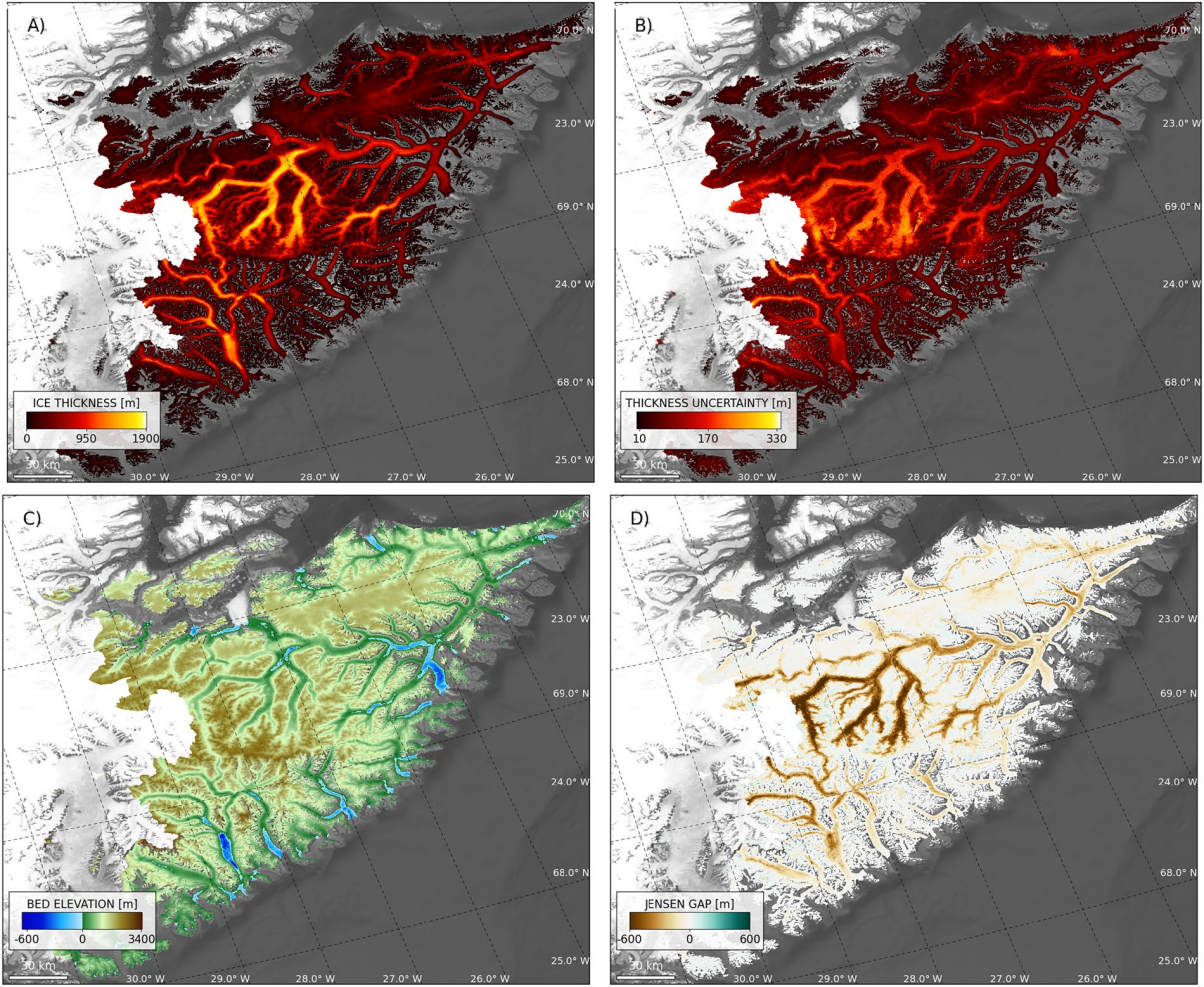
The Geikie Plateau (coastal East Greenland) modeled with IceBoost v2.0. (**A**) ice thickness; (**B**) ice thickness uncertainty; (**C**) bed elevation; (**D**) Jensen Gap.

The TanDEM-X 30m EDEM was produced within 2010-2014, therefore all geodetic features (elevation and its gradients) inherit this time tag^27–29^. The SAR-derived ice velocity product is tagged to 2017-2018^1^. The glacier polygons used to train the model are those from the Randolph Glacier Inventory v6.0^31^. Most glacier polygons in RGI v6.0 and the most recent RGI v7.0 are time tagged between 2000-2010 (Fig. 2 in^31^).

The distance to the ocean was inferred using the ocean vectors digitalized in the GSHHG v2.3.7 product, tagged in June 2017. The glacier-integrated mass balance dataset of Hugonnet *et al*.^7^ is tied to 2000-2019, while the RACMO2 products used in Greenland and Antarctica were averaged over 1961–1990 and 1979–2021, respectively. The temperature-above-2-meter input was calculated by combining ERA5 with ERA5-Land. Both products are considered by averaging all monthly maps over 2000-2010 to generate one single global temperature field. Finally, the ice thickness data used to train the model has a lower cutoff at 2005, with much more measurements acquired after 2010.

To conclude, we estimate our product to be tagged to 2010-2018.

### Ice volumes and sea level equivalent (SLE)

Glacier areas were calculated as ellipsoidal geodesic areas on the WGS84 reference ellipsoid from RGI polygons. A regular 100 × 100 m point grid was then generated within each polygon. Points were accepted only if located within the external glacier boundary and outside any internal nunataks. An equivalent pixel area (hereafter referred to as pixel area) was defined as:

1 $${A}_{n}=\frac{{A}_{{\rm{glacier}}}}{N},$$

where *A*_glacier_ is the glacier area and *N* is the number of accepted grid points within the polygon. Note that *A*_*n*_ generally differs from the nominal 100 × 100 m^2^ grid spacing area, as it accounts for boundary truncation and the exclusion of non-glacier areas (i.e. nunataks). We ensured that the number of accepted grid points *N* exceeded 10^3^. As a result, for small glaciers, the grid spacing was iteratively reduced from the nominal 100 m resolution until this criterion was met. For each glacier, the ice volume *V*_ice_ was then calculated as:

2 $${V}_{{\rm{ice}}}=\sum _{n}{H}_{n}\,{A}_{n},$$

where *H*_*n*_ = *f*(*X*_*n*_) is the modeled ice thickness at grid point *n*, *X*_*n*_ is the feature vector, *A*_*n*_ is the pixel area, and the summation is performed over all accepted grid points.

An upper-bound uncertainty on the ice volume was calculated as:

3 $$\begin{array}{r}{\sigma }_{{V}_{{\rm{ice}}}}=\sum _{n}{\sigma }_{{H}_{n}}{A}_{n},\end{array}$$

assuming perfect positive correlation between pixel errors (*ρ* = 1). The thickness uncertainties ${\sigma }_{{H}_{n}}$ are described in the Technical Validation section.

The ice volume sea-level equivalent (SLE, in mm) was calculated from the ice volume above flotation *V*_af_:

4 $${V}_{{\rm{a}}{\rm{f}}}=\sum _{n}\,max\,[{H}_{n}+min\,({b}_{n},0)\frac{{\rho }_{{\rm{o}}{\rm{c}}{\rm{e}}{\rm{a}}{\rm{n}}}}{{\rho }_{{\rm{i}}{\rm{c}}{\rm{e}}}},0\,]\cdot {A}_{n},$$

5 $${\rm{SLE}}=\frac{{V}_{{\rm{af}}}}{{A}_{{\rm{ocean}}}}\frac{{\rho }_{{\rm{ice}}}}{{\rho }_{{\rm{ocean}}}}\cdot 1{0}^{6}$$

In Eq. (4), *b* is the bed elevation relative to the EIGEN-6C4 geoid, computed as *b* = *z* − *N* − *H*, where *z* is the DEM surface elevation referenced to the WGS84 ellipsoid, *N* is the geoid height, and *H* is the ice thickness. We assume *ρ*_ice_ = 917 kg m^−3^ and *ρ*_ocean_ = 1027 kg m^−3^ as the densities of ice and seawater, respectively, and *A*_ocean_ = 3.618 ⋅ 10^8^ km^2^ as the global ocean area. Steric and isostatic effects are neglected. Our SLE formulation is the same as Millan *et al*.^1^, while Farinotti *et al*.^2^ compute SLE based on ice volume above sea level rather than above flotation.

The quantity

$$H+\min (b,0)\frac{{\rho }_{{\rm{ocean}}}}{{\rho }_{{\rm{ice}}}}$$

represents the height above flotation, denoted *H*_af_. We assume its uncertainty to be equal to the ice thickness uncertainty:

6 $${\sigma }_{{H}_{{\rm{af}}}}=\left\{\begin{array}{ll}{\sigma }_{H}, & \,{\rm{if}}\,{H}_{{\rm{af}}} > 0,\\ 0, & \,{\rm{otherwise.}}\end{array}\right.$$

Therefore, the SLE uncertainty for each glacier is given by:

7 $${\sigma }_{{V}_{{\rm{af}}}}={\sum }_{n}{\sigma }_{{H}_{{\rm{af}}}}\cdot {A}_{n},$$

8 $${\sigma }_{{\rm{SLE}}}=\frac{{\sigma }_{{V}_{{\rm{af}}}}}{{A}_{{\rm{ocean}}}}\frac{{\rho }_{{\rm{ice}}}}{{\rho }_{{\rm{ocean}}}}\cdot 1{0}^{6}\,,$$

In other words, only regions with positive height above flotation contribute to the SLE uncertainty.

The regional ice volumes were obtained by summing individual glacier volumes within each region (Eq. (2), Table 3). Regional volume uncertainties were computed assuming perfect positive correlation between all glacier contributions (*ρ* = 1), consistent with^1,2^, yielding ${\sigma }_{{V}_{{\rm{rgi}}}}={\sum }_{i}{\sigma }_{{V}_{i}}$, where *i* indexes glaciers within a region. This approach provides a conservative upper bound on regional uncertainty. Regional SLE uncertainties were derived analogously, assuming perfect positive correlation among glacier SLE contributions within each region. Regional ice volumes and SLEs (Table 3) are generally consistent across the three models within one standard deviation, with few exceptions (e.g. the Southern Andes, RGI 17). However, individual glacier thickness distributions can differ substantially among models (Supplementary Information, Section S5). A comparison of regional ice volumes and SLE between RGI v6.0 and v7.0 using IceBoost v2.0 is provided in Supplementary Information Table S3.

**Table 3:** Regional ice volumes and sea level equivalent (SLE), referenced to RGI v6.0.

Region	No. glaciers	Area (10^3 ^km^2^)	Ice volume (10^3 ^km^3^)			SLE (mm)		
			IceBoost v2.0	Millan 2022	Farinotti 2019	IceBoost v2.0	Millan 2022	Farinotti 2019
01 Alaska	27108	86.7	16.9 ± 3.2	18.0 ± 5.2	19.0 ± 5.0	41.0 ± 8.5	43.4 ± 14.3	43.3 ± 11.2
02 Western Canada and US	18855	14.5	1.5 ± 0.4	1.2 ± 0.4	1.1 ± 0.3	3.7 ± 1.1	3.0 ± 1.0	2.6 ± 0.7
03 Arctic Canada North	4556	105	24.4 ± 6.3	25.4 ± 7.2	28.3 ± 7.4	58.3 ± 15.2	59.9 ± 19.2	64.8 ± 16.8
04 Arctic Canada South	7415	40.9	7.1 ± 1.8	7.0 ± 2.1	8.6 ± 2.2	17.2 ± 4.5	17.7 ± 5.8	20.5 ± 5.3
05 Greenland Periphery	19306	89.7	13.1 ± 4.5	11.8 ± 3.7	15.7 ± 4.1	30.8 ± 10.7	26.9 ± 9.5	33.6 ± 8.7
06 Iceland	568	11.1	4.6 ± 0.7	3.7 ± 0.9	3.8 ± 1.0	11.2 ± 1.8	9.4 ± 2.6	9.1 ± 2.4
07 Svalbard	1615	34.0	6.7 ± 1.5	7.0 ± 2.3	7.5 ± 1.9	15.5 ± 3.8	15.4 ± 5.7	17.3 ± 4.5
08 Scandinavia	3417	2.9	0.35 ± 0.09	0.29 ± 0.10	0.30 ± 0.08	0.84 ± 0.22	0.73 ± 0.30	0.70 ± 0.20
09 Russian Arctic	1069	51.6	12.8 ± 3.0	15.5 ± 3.9	14.6 ± 3.8	30.3 ± 7.4	33.7 ± 9.6	32.0 ± 8.3
10 North Asia	5151	2.4	0.18 ± 0.07	0.11 ± 0.03	0.14 ± 0.04	0.42 ± 0.15	0.28 ± 0.10	0.30 ± 0.10
11 Central Europe	3927	2.1	0.11 ± 0.05	0.12 ± 0.05	0.13 ± 0.03	0.25 ± 0.13	0.29 ± 0.10	0.30 ± 0.10
12 Caucasus and Middle East	1888	1.3	0.07 ± 0.03	0.05 ± 0.03	0.06 ± 0.02	0.17 ± 0.06	0.14 ± 0.10	0.20 ± 0.10
13 Central Asia	54429	49.3	3.8 ± 2.2	4.4 ± 1.7	3.27 ± 0.85	9.1 ± 5.4	11.2 ± 4.9	7.9 ± 2.0
14 South Asia West	27988	33.6	3.9 ± 1.5	3.8 ± 1.5	2.87 ± 0.74	9.3 ± 3.8	9.6 ± 4.2	6.9 ± 1.8
15 South Asia East	13119	14.7	1.0 ± 0.4	1.2 ± 0.5	0.88 ± 0.23	2.4 ± 1.1	3.1 ± 1.4	2.1 ± 0.5
16 Low Latitudes	2939	2.3	0.11 ± 0.07	0.07 ± 0.04	0.10 ± 0.03	0.27 ± 0.16	0.18 ± 0.10	0.20 ± 0.10
17 Southern Andes	15908	29.4	7.2 ± 1.4	5.90 ± 1.60	5.3 ± 1.4	17.4 ± 3.4	14.6 ± 4.4	12.8 ± 3.3
18 New Zealand	3537	1.2	0.09 ± 0.03	0.07 ± 0.03	0.07 ± 0.02	0.22 ± 0.08	0.18 ± 0.10	0.20 ± 0.10
19 Antarctic and Subantarctic	2752	133	45.6 ± 10.3	35.1 ± 9.1	46 ± 12	77.5 ± 24.6	61.3 ± 16.0	69.4 ± 18.0
Global	215547	706	150 ± 38	141 ± 40	158 ± 41	326 ± 92	311 ± 100	324 ± 84

Comparison between IceBoost v2.0 (this work), Millan *et al*.^1^ and Farinotti *et al*.^2^.

## Data records

The individual-glacier dataset^16^ consists of n = 215,547 and n = 274,531 glacier .tif files, respectively for RGI v6.0 and RGI v7.0. The dataset is available on Zenodo at 10.5281/zenodo.17724512. Each .tif file includes 5 arrays:

- The modeled ice thickness.
- The modeled ice thickness uncertainty.
- The surface elevation.
- The geoid elevation.
- The Jensen Gap.

The surface elevation is taken from the TanDEM-X 30 m Edited EDEM v.1, while the geoid is taken from the EIGEN-6C4 gravity field model (9 km resolution). The map projection is UTM, except for: Greenland (RGI 5, projection 3413), and Antarctica (RGI 19, south of 60^∘^ S, projection 3031). Glaciers in RGI 19 north of 60^∘^ S are released in UTM projection. All layers are release with horizontal resolution of 100 m, except for small glaciers, where is a finer grid is produced. The file attributes include glacier RGI codes, glacier names (if available), and a representative interior point (latitude-longitude) useful for geographical filtering. They also include glacier area *km*^2^, ice volumes (total and below sea level) with associated uncertainties (*km*^3^), in-situ measurements within the glacier (latitude, longitude, and ice thickness), if available, as well as map spatial resolution and projection.

Note that the elevation tiles over the Jan Mayen island are taken from TanDEM-X EDEM v.2, which is not yet publicly available. They are available in the tif files at 100 meters, and can be made available on their original resolution upon request to the TanDEM-X science coordination team.

The regional mosaic dataset^17^ consists of spatially merged ice thickness distributions. It is available on Zenodo at https://zenodo.org/records/20463551. For each region, mosaics are generated within individual UTM zones by merging all glaciers located in the corresponding zone. The data layers are identical to those provided in the individual-glacier product. Associated attributes include regional mosaic area, ice volume (with uncertainty), and ice volume below sea level (with uncertainty). Where available, ground truth ice thickness measurements are also included. The product is generated at a uniform 100 m spatial resolution and is gap-free.

We also release the training dataset (ice thickness + input features) and the trained model (iceboost-v2.0) on Zenodo at 10.5281/zenodo.17724512.

## Usage Notes

A Web visualizer is available at: https://nmaffe.github.io/iceboost_webapp/. Individual glacier files are available for download through the portal. For bulk downloads, users are referred to the datasets hosted on Zenodo^16,17^.

## Data Overview

A representative example of the deposited product is shown in Figure 2 for the Geikie Plateau (East Greenland): modeled ice thickness (A), modeled ice thickness uncertainty (B), bed elevation derived from the difference between surface elevation and ice thickness (C), and the Jensen Gap (D). Thickness uncertainty and the Jensen Gap are discussed in the Technical Validation section.

**Fig. 3:**
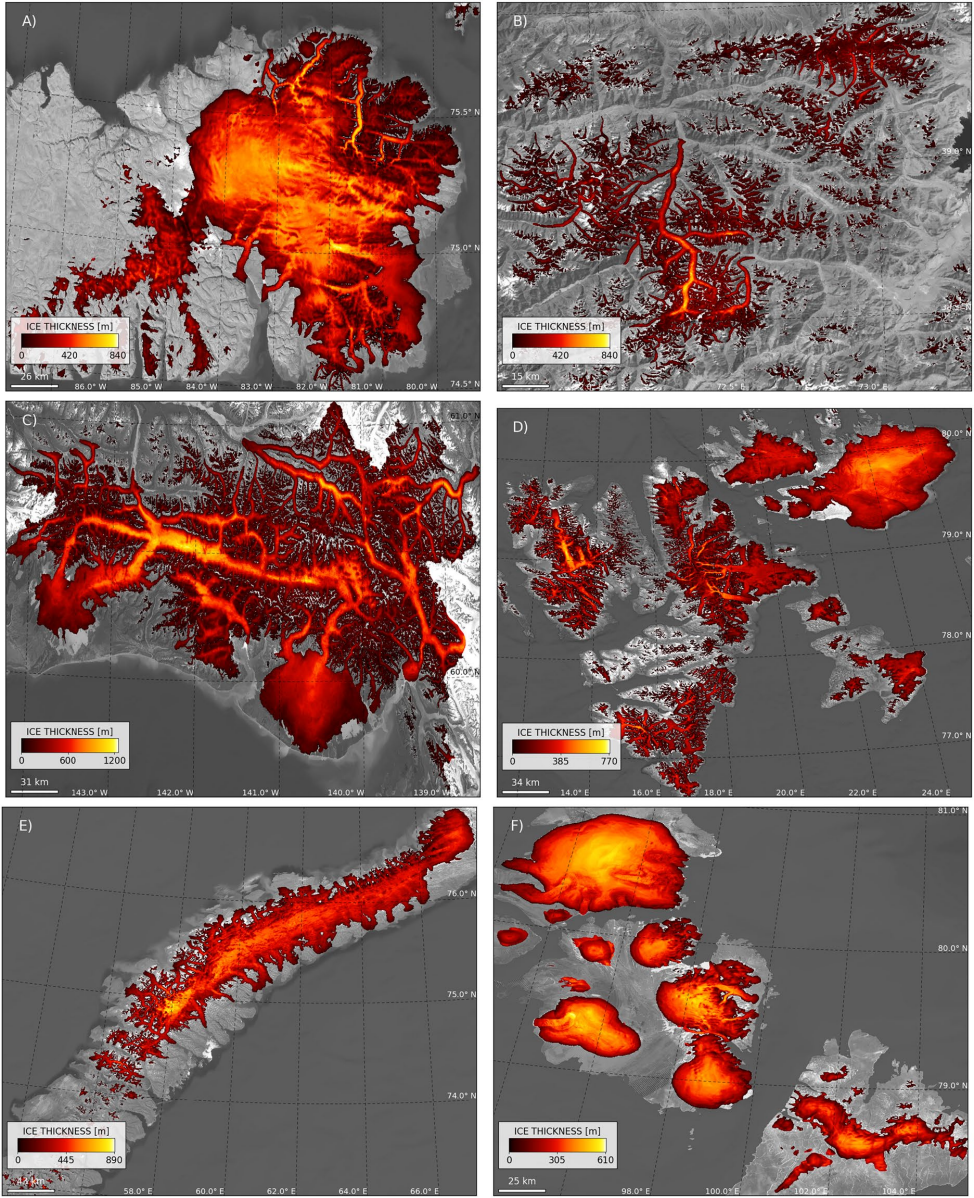
Modeled regions, glaciers and ice caps. (**A**) Devon ice cap (Arctic Canada North); (**B**) Fedchenko glacier and Yazgulem Range (Pamir Mountains, Tajikistan); (**C**) Bagley Icefield and Bering glacier system (Alaska); (**D**) Svalbard archipelago; (**E**) Novaya Zemlya (Russian Arctic); (**F**) Severnaya Zemlya (Russian Arctic). Zoom in for best view.

**Fig. 4:**
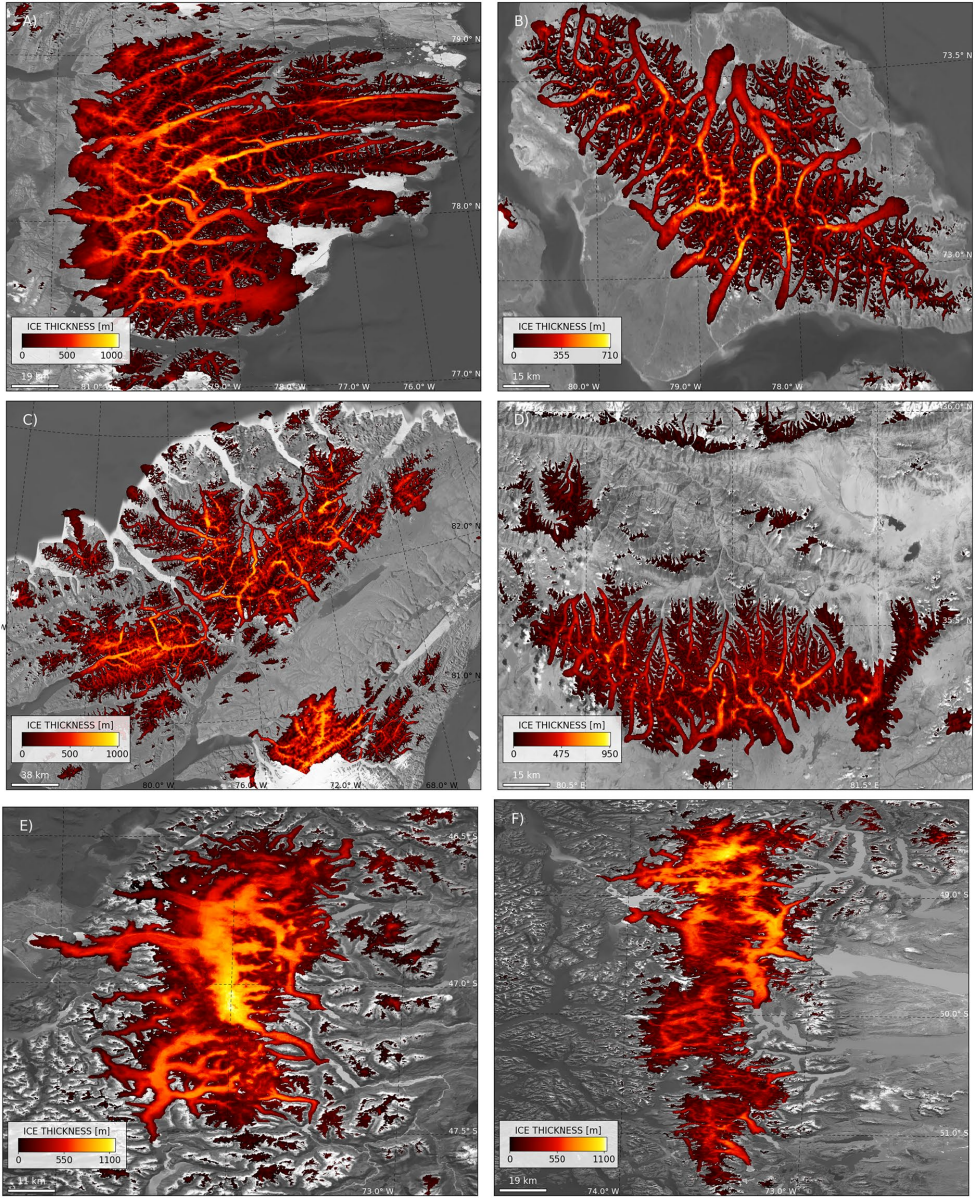
Modeled regions, glaciers and ice caps. (**A**) Prince of Wales icefield (Arctic Canada North); (**B**) Bylot Islands (Arctic Canada South); (**C**) North Ellesmere Icefield (Arctic Canada North); (**D**) West Kulun mountain range (China); (**E**) North Patagonian Icefield (Chile); (**F**) South Patagonian Icefield (Chile). Zoom in for best view.

Figures 3 and 4 show ice thickness maps from major glacierized regions worldwide.

## Technical Validation

The following sections present model input and output uncertainties.

### Elevation, slope and curvature uncertainties

The absolute height accuracy of the TanDEM-X Edited DEM, 30m is typically < 10 m, with relative vertical accuracy (LE90) typically under 2 meters on gentle slopes (slope < 20%), and under 4 meters on steeper slopes (slope > 20%). We set the elevation error Δz = 2 m or 4 m depending on the slope value.

The uncertainty on the slope features, calculated using central differences, ${\rm{s}}=\frac{\Delta {\rm{z}}}{2\Delta {\rm{x}}}$, is calculated as ${{\rm{\sigma }}}_{{\rm{s}}}=\sqrt{{4}^{2}+{4}^{2}}/(2\Delta {\rm{x}})$, where σ_z_ = 4 m everywhere as an upper bound, and Δx is the horizontal step, which can have values ranging from 50 meters to 2 kilometers depending on the slope feature considered (i.e. s_50_, s_100_, s_200_, etc). For example, ${{\rm{\sigma }}}_{{{\rm{s}}}_{50}}=\sqrt{{4}^{2}+{4}^{2}}/(2\cdot 50)=0.056$.

The curvature is calculated as the Laplacian, ${\rm{c}}{\rm{u}}{\rm{r}}{\rm{v}}={\nabla }^{2}{\rm{z}}=\frac{{\partial }^{2}{\rm{z}}}{\partial {{\rm{x}}}^{2}}+\frac{{\partial }^{2}{\rm{z}}}{\partial {{\rm{y}}}^{2}}$. It is approximated using the 5-point Laplacian stencil on a 3 × 3 grid, curv ~ (z_i+1,j_ + z_i−1,j_ + z_i,j+1_ + z_i,j−1_ − 4z_i,j_)/Δx. The error on the curvature is calculated as ${{\rm{\sigma }}}_{{\rm{c}}{\rm{u}}{\rm{r}}{\rm{v}}}=\sqrt{20{{\rm{\sigma }}}_{{\rm{z}}}^{2}}/\Delta {{\rm{x}}}^{2}=2\sqrt{5}/\Delta {{\rm{x}}}^{2}$ (assuming σ_*z*_ = 4 m everywhere as an upper bound). For example, ${{\rm{\sigma }}}_{{{\rm{c}}}_{50}}=2\sqrt{5}/(5{0}^{2})$. An extra factor 100 usually appears as the curvature is reported in units of 1/100 m.

To summarize:

9 $${{\rm{\sigma }}}_{{\rm{z}}}=2-4\,{\rm{m}}$$

10 $${{\rm{\sigma }}}_{{\rm{s}}}=2\sqrt{2}/\Delta {\rm{x}}$$

11 $${{\rm{\sigma }}}_{{\rm{c}}{\rm{u}}{\rm{r}}{\rm{v}}}=2\sqrt{5}{{\rm{\sigma }}}_{{\rm{z}}}/\Delta {{\rm{x}}}^{2}\,{{\rm{m}}}^{-1}$$

### Distances uncertainties

The distance from ice-free pixels uncertainty, σ_noice_, is calculated from the RGI glacier outlines, which are derived mostly from satellite imagery: Landsat TM/ETM+ (30 m pixels), ASTER (15 m pixels), plus other sources in regions where higher-resolution imagery or mapping exists. The spatial resolution therefore varies. We estimate the spatial resolution to be that of the input imagery (approximately 15-30 m), and the associated error to be on the order of one pixel (approximately 30 m). We note, however, that additional and potentially significant sources of uncertainty include rapid glacier retreat in many regions, as well as mapping and georeferencing errors. These factors contribute to uncertainty in distances to glacier margins (or to nunataks within them). We therefore adopt an increased uncertainty estimate of 100 m for this variable.

The distance from the ocean uncertainty σ_ocean_ is calculated using the shorelines product (ocean/land interface in GSHHG), with a precision in the range of 50-500m^34^. This variable is most important for maritime glaciers, near the ocean (rather than continental glaciers located very far from the oceans). We set the error of this product to be 100 m.

12 $${\sigma }_{noice}=100\,m$$

13 $${\sigma }_{ocean}=100\,m$$

### Glacier length uncertainty

The glacier length (*lmax*), calculated using the convex hull, is the as maximum distance across the glacier. The error is taken as the 5%, to account for larger uncertainties in irregular shapes for large glaciers:

14 $${\sigma }_{lmax}=0.05\cdot lmax$$

### Ice velocity uncertainty

The uncertainty on the ice velocity is set to 10 m/yr^1,45^ everywhere except for the Greenland periphery, where it is set to 18 m/yr^46^. In the Antarctic periphery and subantarctic glaciers we also use 18 m/yr, a conservative estimate^42^.

15 $${\sigma }_{v}=\left\{\begin{array}{ll}18\,\,{\rm{m/yr}}\,, & \,{\rm{if\; glacier}}\,\in \{\,{\rm{Greenland,\; Antarctica}}\}\\ 10\,\,{\rm{m/yr}}\,, & \,{\rm{else.}}\\ \end{array}\right.$$

### Temperature uncertainty

The temperature above 2 m (t2m) is calculated by averaging N=120 monthly maps (for the 2000-2010 period). If monthly consecutive maps can be modeled to have a lag-1 autocorrelation *ρ* (AR(1)), where *ρ* is the positive correlation between consecutive monthly maps, the effective sample size is approximately

16 $${N}_{eff}\approx N\frac{1-\rho }{1+\rho }$$

For non-correlated maps, *ρ*=0, *N*_*eff*_ = *N* = 120. For a positive and moderately-high correlation, *ρ* = 0.8, each map provides less information and the effective sample size becomes ${N}_{eff}=120\frac{1-0.8}{1+0.8}\approx 13.3$.

If further assuming that each monthly map has a realistic error *σ* ≈ 1 K, the random error of the 10 year average becomes

17 $${\sigma }_{random}=\frac{\sigma }{{N}_{eff}}=\frac{1}{\sqrt{13.3}}\approx 0.3\,K$$

We should now also consider a systematic bias *b* due to a finite grid of the satellite product, impacting in particular regions with complex and poorly resolved mountainous terrain. We set such bias everywhere to be *b* = 1 K. We calculate the final temperature uncertainty by accounting for both independent errors:

18 $${\sigma }_{t2m}=\sqrt{{\sigma }_{random}^{2}+{b}^{2}}\approx \sqrt{0.{3}^{2}+1}\approx 1\,K.$$

The t2m uncertainty is approximated as 1 K everywhere. Such uncertainty is likely realistic for high mountainous regions, and overestimated over flatter and well-behaving terrain.

### Surface mass balance uncertainty

RACMO2.3p2 modeled surface mass balance is used in the peripheries of the Greenland (resolution 1 km) and Antarctica (resolution 2 km), both featuring complex-terrain zones, spatial gradients, and poorly resolved orographic effects. Monthly maps are averaged over 1961-1990 for Greenland and 1979-2021 for Antarctica to produce a single multi-decadal mean map over the two ice sheets. Such a long temporal period would reduce the influence of interannual variability (much like temperature) on the mean, and it is not considered here, but leaving the dominant source of uncertainty to RACMO2.3p2 model’s systematic bias, estimated as 10% the averaged accumulation values. Such value is inferred from RACMO2.3p2’s observed difference between the coarse (27km)-to-downscaled (2 km) products in the Antarctic peninsula^38^, which gives an insight to such resolution-driven bias.

For all glaciers outside the ice sheet peripheries, we use the same SMB-to-elevation lapse rate method in IceBoost v1.1^18^. We use regional values of $\bar{s}=dSMB/dz$ and $\bar{q}=SMB(z=0)$ for every glacier in each regions. No robust a priori rationale can be used to formulate the uncertainties on these pairs. A sensitivity test was carried out in^18^ to assess how much modeled integrated glacier volumes would change by changing these values, and the authors found a limited sensitivity to this parametrization. We formulate the uncertainties on these two parameters to be 10%: ${\sigma }_{\bar{s}}=0.1\bar{s}$, ${\sigma }_{\bar{q}}=0.1\bar{q}$. To summarize, in the ice sheet peripheries:

19 $$\left\{\begin{array}{l}{\rm{SMB}}={\rm{RACMO}}2.3{\rm{p}}2\\ {\sigma }_{{\rm{SMB}}}=0.1\cdot {\rm{SMB}}\end{array}\right.$$

For all other glaciers:

20 $$\left\{\begin{array}{l}{\rm{SMB}}=\bar{{\rm{q}}}+\bar{{\rm{s}}}{\rm{z}}\\ {\sigma }_{{\rm{SMB}}}=0.1\bar{{\rm{q}}}+0.1\bar{{\rm{s}}}{\rm{z}}\end{array}\right.$$

where z is elevation in meters; $\bar{{\rm{s}}}$ has units of mm w.e.yr^−1^m^−1^; $\bar{{\rm{q}}}$ has units of mm w.e. yr^−1^; SMB has units of mm w.e.yr^−1^m^−1^. The regional ($\bar{{\rm{q}}}$, $\bar{{\rm{s}}}$) values can be found in Appendix A, Table A1 of Maffezzoli *et al*.^18^.

### Ice thickness uncertainty via Monte Carlo simulations

To estimate uncertainties in the modeled glacier ice thickness maps, we perform a Monte Carlo (MC) perturbation analysis. For each glacier, we collect the feature set *X*, assigning an uncertainty *σ* to each input feature (e.g., elevation, slope, velocity), as discussed in the previous sections. We then generate n=50 random realizations of the input features *X*, perturbing each set as *X* + *ϵ*, where $\epsilon  \sim {\mathcal{N}}\,(0,{\sigma }^{2})$ and ${\mathcal{N}}$ is a Gaussian.

IceBoost is evaluated for each realization, *f*(*X* + *ϵ*), producing an ensemble of ice thickness maps. The standard deviation of this ensemble, *σ*_*MC*_ = *σ* (*f*(*X* + *ϵ*)), contains two contributions: i) the aleatoric variability of predictions due to imperfect knowledge of the inputs (introduced through perturbations), and ii) the epistemic uncertainty arising from differences between the two learning algorithms (XGBoost and CatBoost). While the first component vanishes as input uncertainties tend to zero, the second does not and quantifies the systematic disagreement between the XGBoost and CatBoost models. We take *σ*_*MC*_(*x*, *y*) as the ice thickness uncertainty, i.e. *σ*_*H*_(*x*, *y*) = *σ*_*MC*_(*x*, *y*). An example of the *σ*_*H*_(*x*, *y*) map for the Geikie Plateau (East Greenland) is shown in Fig. 2. The ice thickness uncertainty maps are released for each glacier. In general, higher uncertanity corresponds to higher ice thickness. The main reason for that is that higher ice thickness corresponds to regions where slope are minimum. Uncertainty induced in slope variables (via perturbations) has a significant impact on predicted ice thickness. In mountainous terrain (higher slope, thinner ice), perturbations have a weaker effect, resulting in a narrower range of estimates and thus lower *σ*_*MC*_(*x*, *y*). Such effect is further investigated in the following section.

### Model non-linearity and Jensen Gap

We aim to identify where IceBoost behaves nonlinearly and how input perturbations (i.e., uncertainties in the predictors) affect the modeled ice thickness. According to Jensen’s inequality, for a random variable *X* and a function *f*,

21 $$\begin{array}{l}{\mathbb{E}}[f(X)]-f({\mathbb{E}}[X]) > 0\,\mathrm{if}\,f\,\mathrm{is}\,\mathrm{convex},\\ {\mathbb{E}}[f(X)]-f({\mathbb{E}}[X]) < 0\,\mathrm{if}\,f\,\mathrm{is}\,\mathrm{concave}.\end{array}$$

and the quantity

22 $${\mathcal{J}}(f,X)={\mathbb{E}}[f(X)]-f({\mathbb{E}}[X])$$

is the Jensen Gap.

${\mathcal{J}}$ depends jointly on the curvature of *f* and the variance of *X*, and it vanishes for linear models. Thus, it provides a measure of how much the model prediction changes as a result of input uncertainty and model curvature. Users should interpret ${\mathcal{J}}(f,X)$ as a diagnostic of model nonlinearity under input uncertainty. It quantifies the discrepancy between the deterministic prediction and the expected model output under perturbed inputs, thereby characterizing the sensitivity of the model predictions to input uncertainty.

In our setting, ${\mathbb{E}}[f(X)]$ is the mean thickness obtained by applying IceBoost to the perturbed features, whereas $f({\mathbb{E}}[X])$ is the thickness predicted from the unperturbed features.

Because IceBoost combines two gradient-boosted decision tree models, it is inherently nonlinear. To probe this nonlinearity, we use the *n* = 50 Monte Carlo perturbations described in the previous section to approximate the distribution of *X* and to compute ${\mathcal{J}}(f,X)$ at each glacier pixel. The resulting Jensen Gap maps identify where - and by how much - input uncertainty would shift the mean modeled thickness. Positive values indicate locally convex model behavior (uncertainty increasing the expected thickness), while negative values indicate concavity (uncertainty decreasing the expected thickness).

**Fig. 5:**
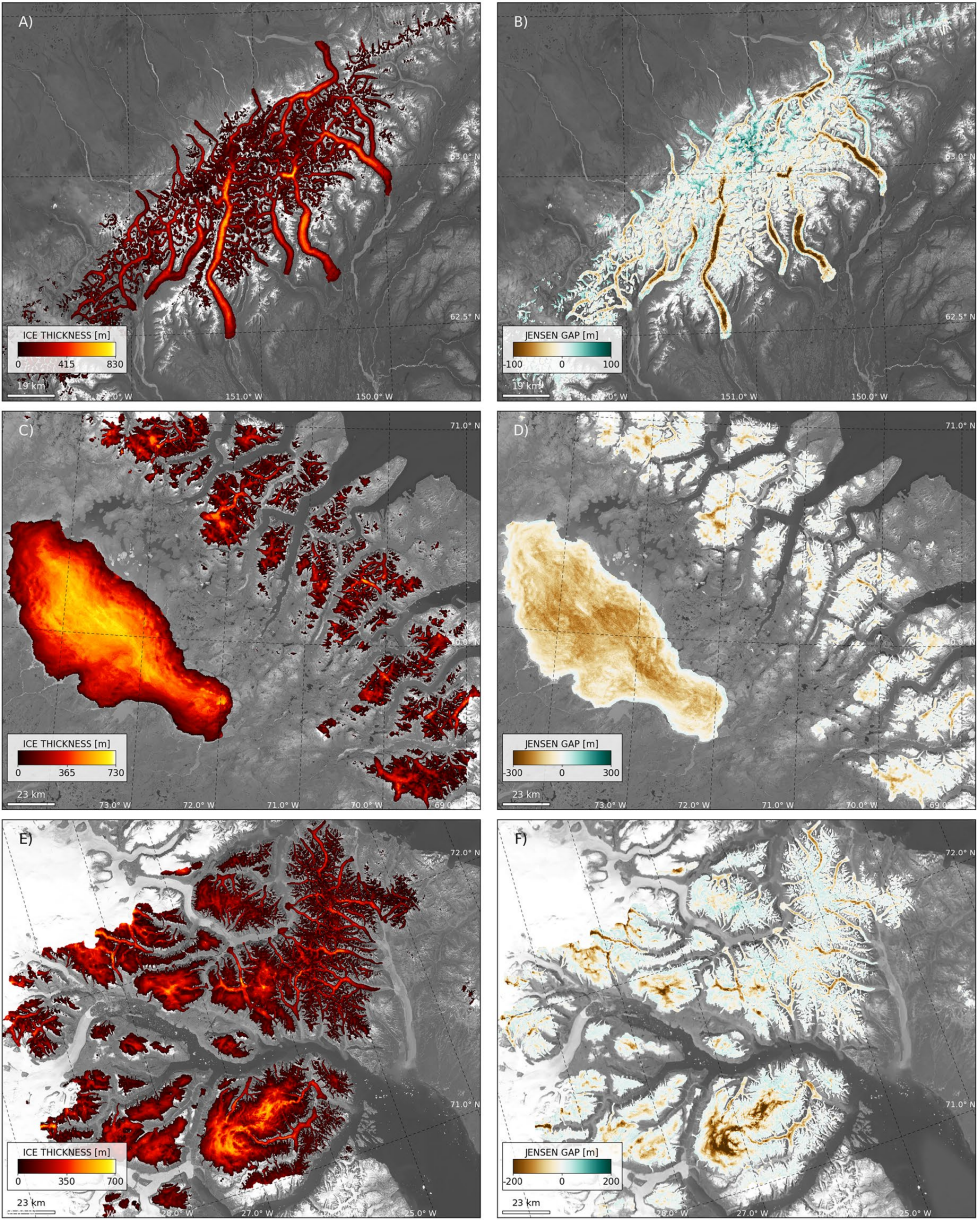
Modeled ice thickness A-C-E and corresponding Jensen Gap B-D-F. (**A**) Ruth glacier and Denali glacier system (Alaska); (**C**) Barnes ice cap and Baffin island glaciers (Nunavut, Canada); (**E**) Renland ice cap and glaciers in the Scoresby Sound system (Eastern Greenland). Negative (positive) Jensen Gap values indicate decreased (increased) modeled ice thickness due to input uncertainty.

We find that ${\mathcal{J}}$ is often negative (Fig. 5). These regions typically correspond to thick ice over low-slope terrain. Previous feature-importance shapely analyses^18^ showed that surface slope is one of the strongest predictors of thickness. Because the model is highly sensitive to slope, perturbing the slope symmetrically has an asymmetric effect on thickness: over low-sloping terrain, a perturbation that increases the slope causes a large drop in predicted thickness. The corresponding perturbation that decreases the slope results in a much smaller gain in thickness. This concave response dominates the sign of the Jensen Gap. Although ${\mathcal{J}}$ reflects the combined effect of all input uncertainties, we suggest that, where ice is thickest, slope variability is the primary driver. The Jensen Gap thus provides an empirical, spatially distributed measure of the curvature of the learned mapping *f*, and highlights where input uncertainties most strongly interact with it.

## Supplementary information

Supplementary Information

## Data Availability

The global individual-glacier and regional mosaic datasets are available on Zenodo at https://zenodo.org/records/17724512 and https://zenodo.org/records/20463551, respectively.

## Code availability

The IceBoost v2.0 model is maintained on GitHub: https://github.com/nmaffe/iceboost/.
