# Supplementary material for: Machine-learned global glacier ice volumes
Source: Sci Data. 2026 Jul 30;13:1104. doi: 10.1038/s41597-026-07744-9 (PMC13424553; doi:10.1038/s41597-026-07744-9)
Supplement: Supplementary file 1 — Supplementary Information [file 41597_2026_7744_MOESM1_ESM.pdf]

Supplementary information

Machine-learned global glacier ice volumes

Niccolò Maffezzoli[1,2,3], Eric Rignot[2,4], Carlo Barbante[1,3], Mathieu Morlighem[5], Troels Petersen[6], and Sebastiano Vascon[1]

[1]Ca' Foscari University of Venice, Venezia, Italy
[2]University of California Irvine, Irvine, USA
[3]Institute of Polar Sciences, National Research Council, Venezia, Italy
[4]Jet Propulsion Laboratory, Pasadena, USA
[5]Department of Earth Sciences, Dartmouth College, Hanover, 03755 NH, United States
[6]Niels Bohr Institute, University of Copenhagen, Copenhagen, Denmark

S1 IceBoost v2.0 hyperparameters

The best hyperparameters are kept the same as IceBoost v1.1, as minimal differences in performance were found in a fresh hyperparameter tuning Bayesian optimization pipeline [1].

Below a list of the hyperparameters to be used for the 3 XGBoost modules and 3 CatBoost modules:

XGBoost Hyperparameter	Value
tree_method	hist
lambda	76.814
alpha	76.374
colsample_bytree	0.9388
subsample	0.741501
learning_rate	0.079244
max_depth	20
min_child_weight	19
gamma	0.18611
num_boost_round	1000 trees
early_stopping_rounds	50
loss	L_2

Table S1: IceBoost v2.0 hyperparameters for all XGBoost modules. For the parameter description, we refer to the XGBoost documentation at https://xgboost.readthedocs.io/en/stable/parameter.html.

CatBoost Hyperparameter	Value
early_stopping_rounds	50
depth	6
learning_rate	0.1
loss	L_2

Table S2: IceBoost v2.0 hyperparameters for all CatBoost modules. For the parameter description, we refer to the CatBoost documentation at https://catboost.ai/en/docs/concepts/parameter-tuning.

S2 Models' performance against ground truth data

The model performance is evaluated following the same cross-validation procedure described in [1]. For each region, the model is trained 100 times (with hyperparameters held fixed), withholding 20% of the regional data for testing and using the remaining global dataset for training. The 20% test subset is randomized at each iteration. The split is applied at the glacier level, ensuring that all points from a given glacier are assigned exclusively to either the training or the testing set. The test set therefore consists of entirely unseen glaciers. Model accuracy is quantified using the root mean squared error (RMSE) and compared against two baseline models ([2, 3]), both bi-linearly interpolated at the target points.

Overall, IceBoost v2.0 achieves performance comparable to IceBoost v1.1, despite being trained with fewer features (albeit with more data by a factor 1.9). In the northern high latitudes (Alaska, the Canadian Arctic and Greenland), IceBoost v2.0 shows ca. 20-45% lower errors compared to the other models. In Greenland, such comparison includes BedMachine-Greenland v5 [4] for glaciers directly connected to the ice sheet (polygons from RGI v.62).

In Svalbard, IceBoost v2.0 and Farinotti's model perform similarly, while in Scandinavia IceBoost v2.0 achieves slightly lower errors than all other models ([2, 3]). At mid-latitudes (Caucasus, Central Europe, North Asia), model performance is generally consistent across approaches; however, only Central Europe provides sufficient data for a robust evaluation (Table S4). In Central Asia (RGI region 13), the dataset is limited to 11 glaciers (5,423 points), and the high standard deviation indicates potential outliers. In the Southern Andes, all models exhibit elevated RMSE, likely due to thickness outliers introduced with newly added data. In this region, IceBoost v2.0 also shows a systematic bias, with predictions lower than the observed thickness.

In Antarctica, the comparison includes BedMachine v3 [5] for both the continental ice and the peninsula, where IceBoost v2.0 performs comparably to BedMachine v3. For peripheral glaciers and Antarctic islands, IceBoost v2.0 achieves similar accuracy to both Millan's and Farinotti's models.

IceBoost v2.0 shows a strong performance and generalization capability over the ice sheet periphery. Achilles' heel of the IceBoost v2.0 is the strong dependence on the quality of the data used for training.

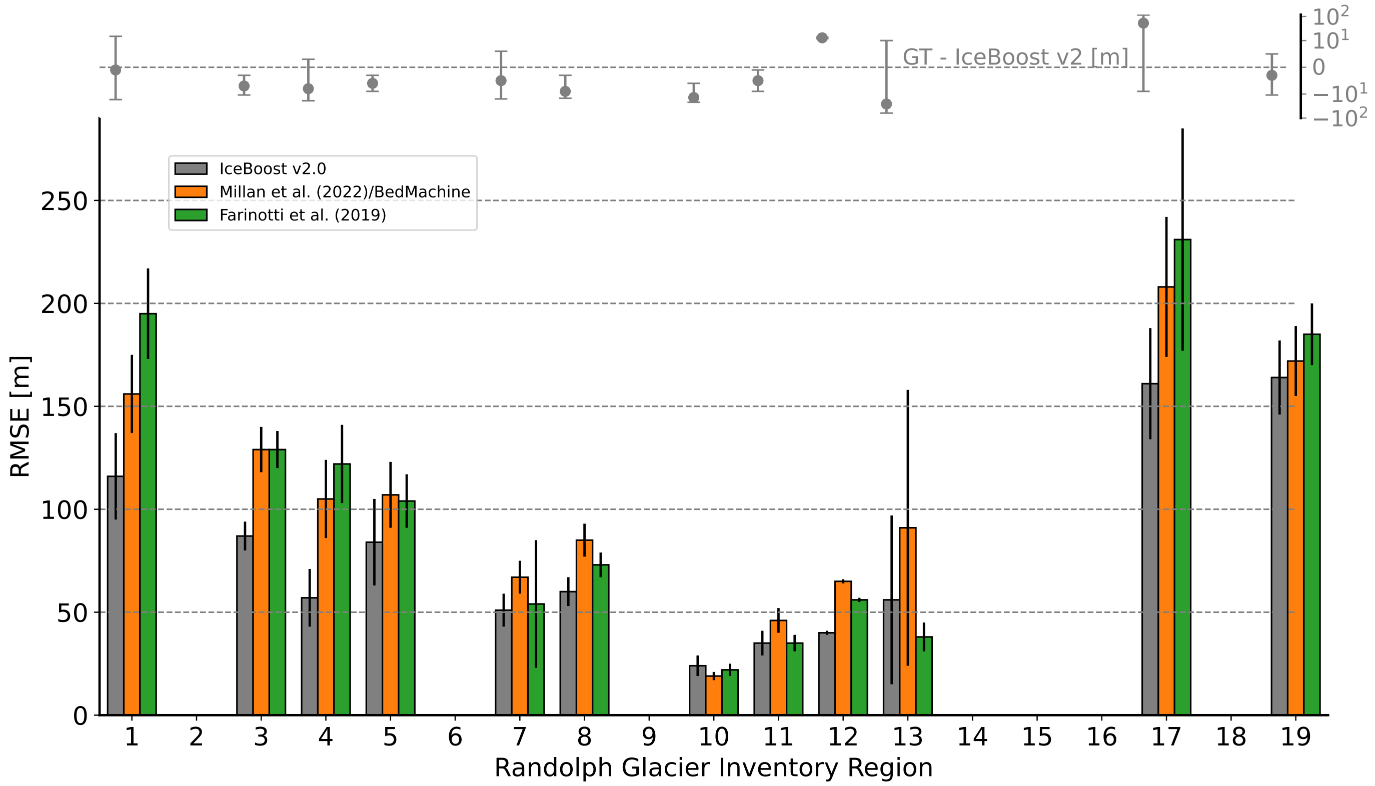

Figure S1: Comparison between the performance of IceBoost v2.0 (this work), Millan et al. (2022, [2]) and Farinotti et al. (2019, [3]). RMSE: root mean squared error, GT: ground truth data.

S3 Regional ice volumes and sea level equivalent - comparison between RGI v6.0 and RGI v7.0

Table S3: Regional ice volumes (unit: $10^3\,km^3$) and sea level equivalent (SLE, unit: mm) estimated by IceBoost v2.0 for RGI v.60 and v.70.

RGI	Region	Ice volume RGI v6.0	Ice volume RGI v7.0	SLE RGI v6.0	SLE RGI v7.0
1	Alaska	16.9 ± 3.2	16.9 ± 3.2	41.0 ± 8.5	39.3 ± 7.8
2	Western Canada and US	1.5 ± 0.4	1.5 ± 0.4	3.7 ± 1.1	3.7 ± 1.1
3	Arctic Canada North	24.4 ± 6.3	24.3 ± 6.2	58.3 ± 15.2	58.0 ± 15.1
4	Arctic Canada South	7.1 ± 1.8	7.1 ± 1.8	17.2 ± 4.5	17.1 ± 4.4
5	Greenland Periphery	13.1 ± 4.5	13.2 ± 4.6	30.8 ± 10.7	31.1 ± 11.0
6	Iceland	4.6 ± 0.7	4.6 ± 0.7	11.2 ± 1.8	11.2 ± 1.8
7	Svalbard	6.7 ± 1.5	6.7 ± 1.5	15.5 ± 3.8	15.5 ± 3.8
8	Scandinavia	0.35 ± 0.09	0.35 ± 0.09	0.84 ± 0.22	0.84 ± 0.22
9	Russian Arctic	12.8 ± 3.0	12.8 ± 3.0	30.3 ± 7.4	30.3 ± 7.4
10	North Asia	0.18 ± 0.07	0.19 ± 0.07	0.42 ± 0.15	0.45 ± 0.16
11	Central Europe	0.11 ± 0.05	0.11 ± 0.05	0.25 ± 0.13	0.26 ± 0.13
12	Caucasus and Middle East	0.07 ± 0.03	0.08 ± 0.03	0.17 ± 0.06	0.19 ± 0.07
13	Central Asia	3.8 ± 2.2	3.8 ± 2.2	9.1 ± 5.4	9.1 ± 5.4
14	South Asia West	3.9 ± 1.5	3.8 ± 1.5	9.3 ± 3.8	8.9 ± 3.7
15	South Asia East	1.0 ± 0.4	1.0 ± 0.5	2.4 ± 1.1	2.4 ± 1.1
16	Low Latitudes	0.11 ± 0.07	0.09 ± 0.06	0.27 ± 0.16	0.21 ± 0.13
17	Southern Andes	7.2 ± 1.4	6.8 ± 1.3	17.4 ± 3.4	16.2 ± 3.1
18	New Zealand	0.09 ± 0.03	0.08 ± 0.03	0.22 ± 0.08	0.18 ± 0.06
19	Antarctic and Islands	45.6 ± 10.3	45.7 ± 10.3	77.5 ± 24.6	78.2 ± 24.7
Total		150 ± 38	149 ± 38	326 ± 92	323 ± 91

S4 Training dataset

Table S4: Training dataset statistics for IceBoost v2.0 following glacier-by-glacier encoding into 100x100 pixel averages. The number of glaciers refers to RGI v.62.

RGI	Region	no. training points	no. glaciers
1	Alaska	30,926	99
2	Western Canada and US	308	2
3	Arctic Canada North	68,413	339
4	Arctic Canada South	28,891	149
5	Greenland Periphery	64,735	568
6	Iceland	0	0
7	Svalbard	45,839	97
8	Scandinavia	38,103	91
9	Russian Arctic	0	0
10	North Asia	704	4
11	Central Europe	25,240	109
12	Caucasus and Middle East	1,354	2
13	Central Asia	5,423	11
14	South Asia West	0	0
15	South Asia East	0	0
16	Low Latitudes	462	3
17	Southern Andes	13,182	40
18	New Zealand	0	0
19	Antarctic Periphery, Peninsula and Islands	55,397	138
Total		378,977	1,652

S5 Modeled ice thickness and comparisons with Millan et al. (2022) and Farinotti et al. (2019)

In Sections S5.1 to S5.6, we present regions of particular interest to the glaciological community: Alaska (Sect. S5.1), the Canadian Arctic (Sect. S5.2), the Russian Arctic (Sect. S5.3), the Greenland periphery (Sect. S5.4), Asia (Sect. S5.5), and the Southern Andes (Sect. S5.6). For each region, we present the most significant glaciated systems and ice caps and discuss differences between IceBoost, the models by Millan et al. [2] and Farinotti et al. [3], and available measurements. We also calculate the the total ice volumes in such regions (Tables S5,S6,S7,S8,S9,S10).

S5.1 Alaska (RGI 01)

Table S5: Alaska ice volumes estimated by different models. All units are $10^3\,\mathrm{km}^3$.

Alaska (RGI 01)	IceBoost v2.0	Millan et al. [2]	Farinotti et al. [3]
Total	16.9 ± 3.2	17.8 ± 4.6	19.0 ± 5.0
- Bering-Malaspina-Seward basins	6.4 ± 1.0	6.7	7.8
- Others	10.5 ± 2.2	11.1	11.2

In the Bering-Malaspina-Seward glacier basins, regions of shallow ice are fairly consistent across all models (Fig. S2). Thick-ice areas show differences. Millan's model shows short-scale fluctuations of shallower and deeper ice. IceBoost and Farinotti's models produce smoother fields. Farinotti models ice is too thick compared to data in the deepest parts of the Bering glacier terminus as well as in the Malaspina terminal lobe. When evaluated against ground-truth data collected in Alaska, IceBoost shows root mean square errors that are 25% and 40% lower (Supp. Info. Fig. S1), indicating that this is the most accurate model for this region. Data and IceBoost indicate that Malaspina, Agassiz, Steller and Bering termini are grounded 100-300 meters below sea level. Yet, IceBoost cannot resolve the bed troughs captured by radar profiles at spatial scales of 100 meters on the Malaspina lobe [6]. All models and data indicate that the Hubbard glacier terminus is grounded up to half a kilometer below sea level in the Disenchantment Bay.

Figure S2: Bering-Malaspina-Seward basin (Alaska, RGI 01). A=IceBoost v2.0 (with overlayed data); B=Millan et al. 2022 [2]; C=Farinotti et al. [3]. Zoom in for best view.

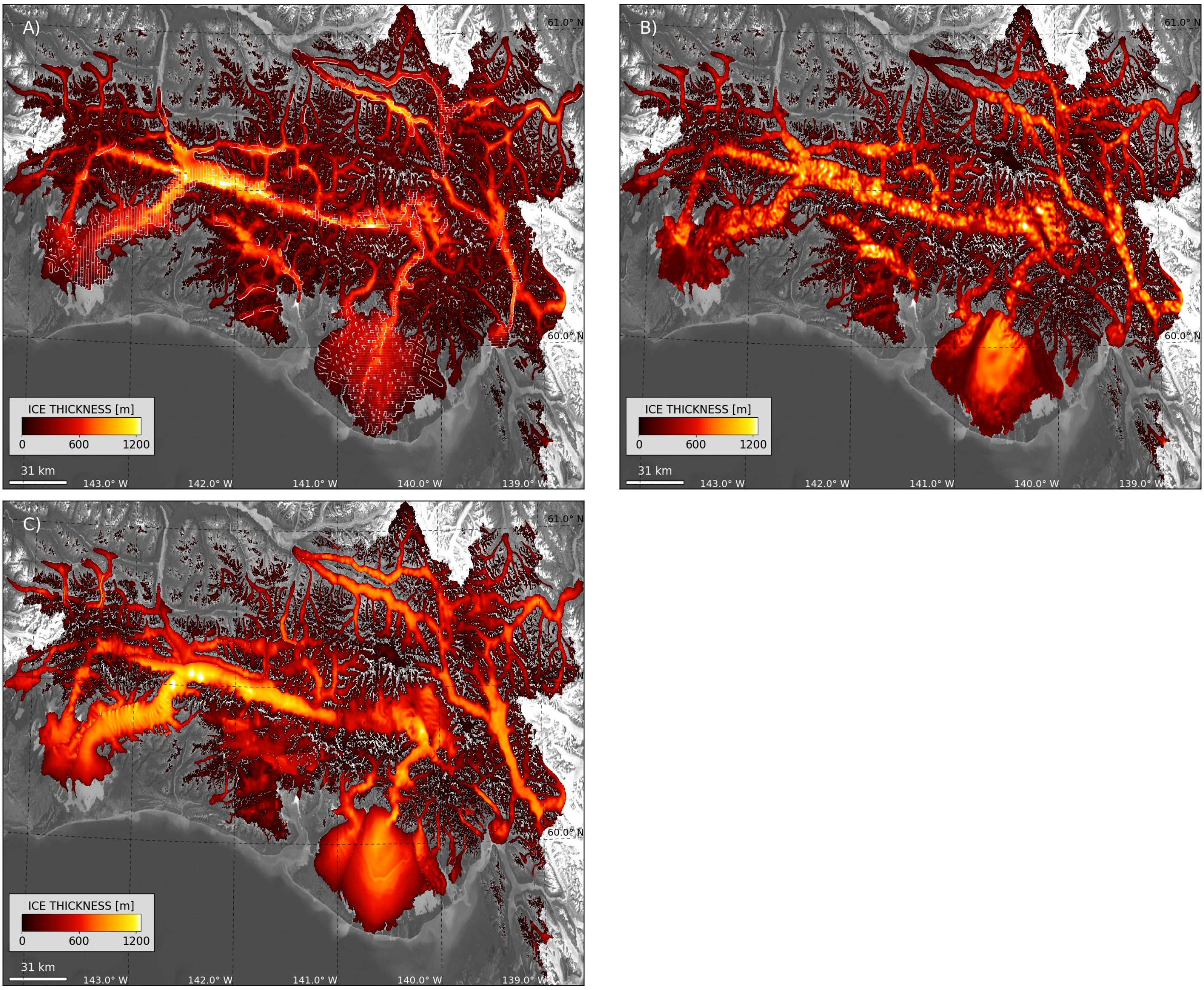

S5.2 Canadian Arctic North and South (RGI 03-04)

Table S6: Canadian Arctic ice volumes estimated by different models. All units are 10^3 km^3.

Canadian Arctic North (RGI 03)	IceBoost v2.0	Millan et al. [2]	Farinotti et al. [3]
Total	24.3 ± 6.2	25.4 ± 7.2	28.3 ± 7.4
- Northern Ellesmere Island	11.137±3.106	10.651	12.180
- Muller icefield	2.28 ± 0.56	2.38	2.49
- Prince Of Wales, SydKap, Manson Icefields	6.269 ± 1.512	6.978	7.871
- Devon ice cap	4.365 ± 0.943	4.87	5.421
- Others	0.249 ± 0.079	0.521	0.338
**Canadian Arctic South (RGI 04)**	**IceBoost v2.0**	**Millan et al. [2]**	**Farinotti et al. [3]**
Total	7.1 ± 1.8	7.0 ± 2.1	8.6 ± 2.2
- Baffin Island North	1.741±0.466	1.509	2.220
- Baffin Island Central	2.893±0.719	2.836	3.313
- Baffin Island South	2.163±0.538	2.308	2.728
- Others	0.303±0.077	0.347	0.339

Comparison with measurements suggests that IceBoost performs best among all models in the Canadian Arctic (RGI regions 3 and 4; Fig. S1), achieving RMSE reductions of 32% in the Northern Canadian Arctic (RGI 3) and 46–53% in the Southern Canadian Arctic (RGI 4), relative to the other models.

In the Northern Ellesmere Island (Fig. S3) deep ice channels are reproduced only by IceBoost.

The Devon ice cap is modeled too thick compared to data by both Millan and Farinotti models, especially in the tidewater glacier terminations on the eastern side of the ice cap (Fig. S3). IceBoost models the southwest arm of the ice cap as thinner than the other reconstructions, by 100-200 meters on average. Very few measurements exist that can confirm this result, as the region remains unsurveyed.

Over the Müller icefield (Fig. S4), IceBoost shows the best agreement with observations. Farinotti's reconstruction is too thick. Millan's model performs better yet shows problems at outlet glaciers. Over the Prince of Wales, SydKap and Manson icefields, IceBoost and Millan's reconstructions works best (Fig. S4). IceBoost (and data) indicates that several glaciers terminating in the Baffin Bay -including Ekblaw, Cadogan, Trinity–Wykeham, and Mittie glaciers - are grounded below sea level for tens of kilometres inland, consistent with published literature [7].

In the Canadian Arctic South (Baffin Island, Figs. S5-S6) measurements acquired over glaciers in the Bylot Island, the Barnes and Penny ice caps indicate that IceBoost has the best agreement with data. IceBoost suggests that no glaciers in the Baffin Island are grounded below sea level.

Figure S3: Canadian Arctic North (RGI 03). Top: Devon Ice Cap. Bottom: Northern Ellesmere Island. A,D=IceBoost v2.0; B,F=Millan et al. 2022 [2]; C,E=Farinotti et al. [3]. Zoom in for best view.

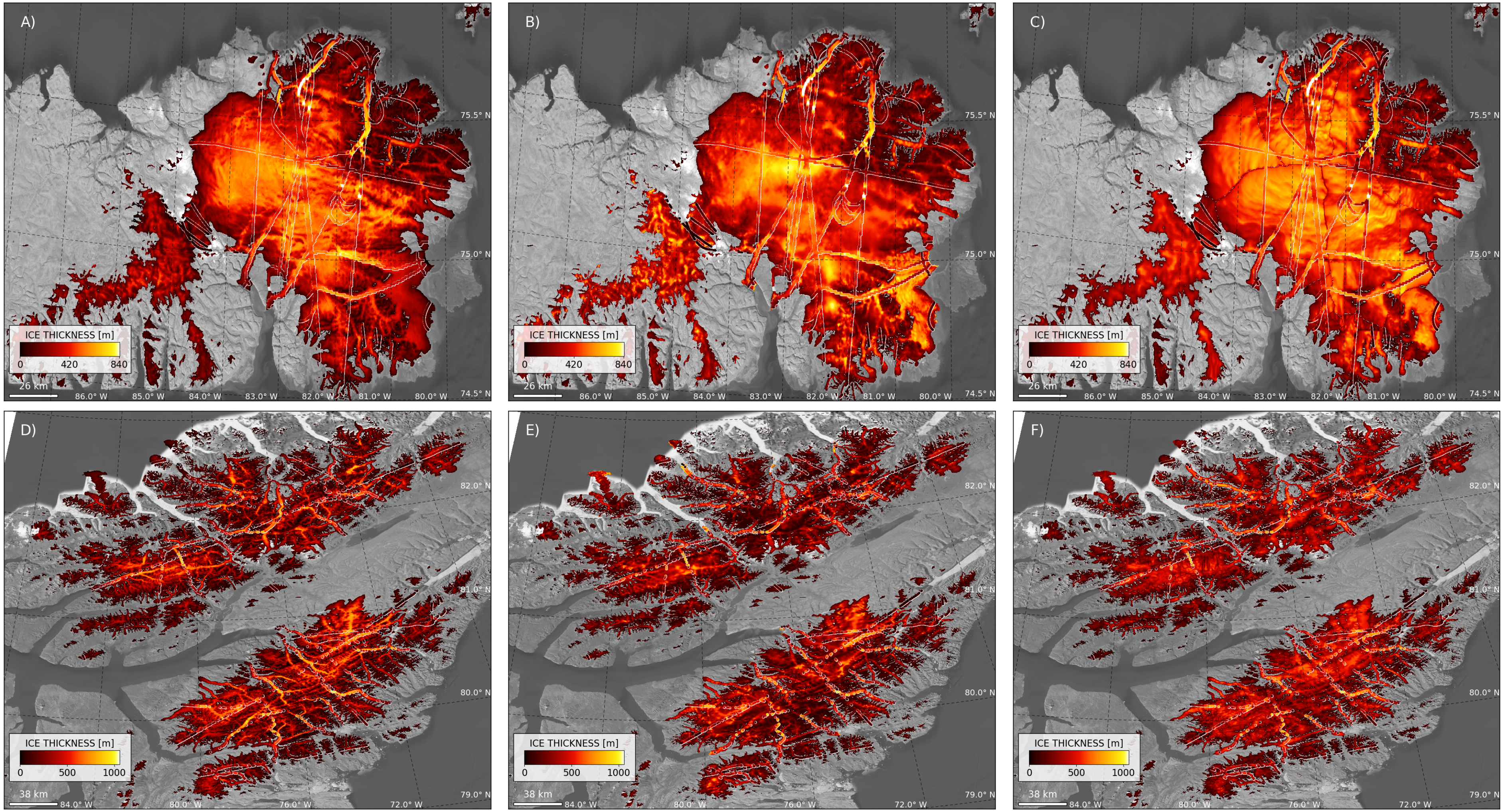

Figure S4: Canadian Arctic North (RGI 03). Top: Muller Icefield; Bottom: Prince of Wales, SydKap and Manson Icefields. A,D=IceBoost v2.0; B,E=Millan et al. 2022 [2]; C,F=Farinotti et al. [3]. Zoom in for best view.

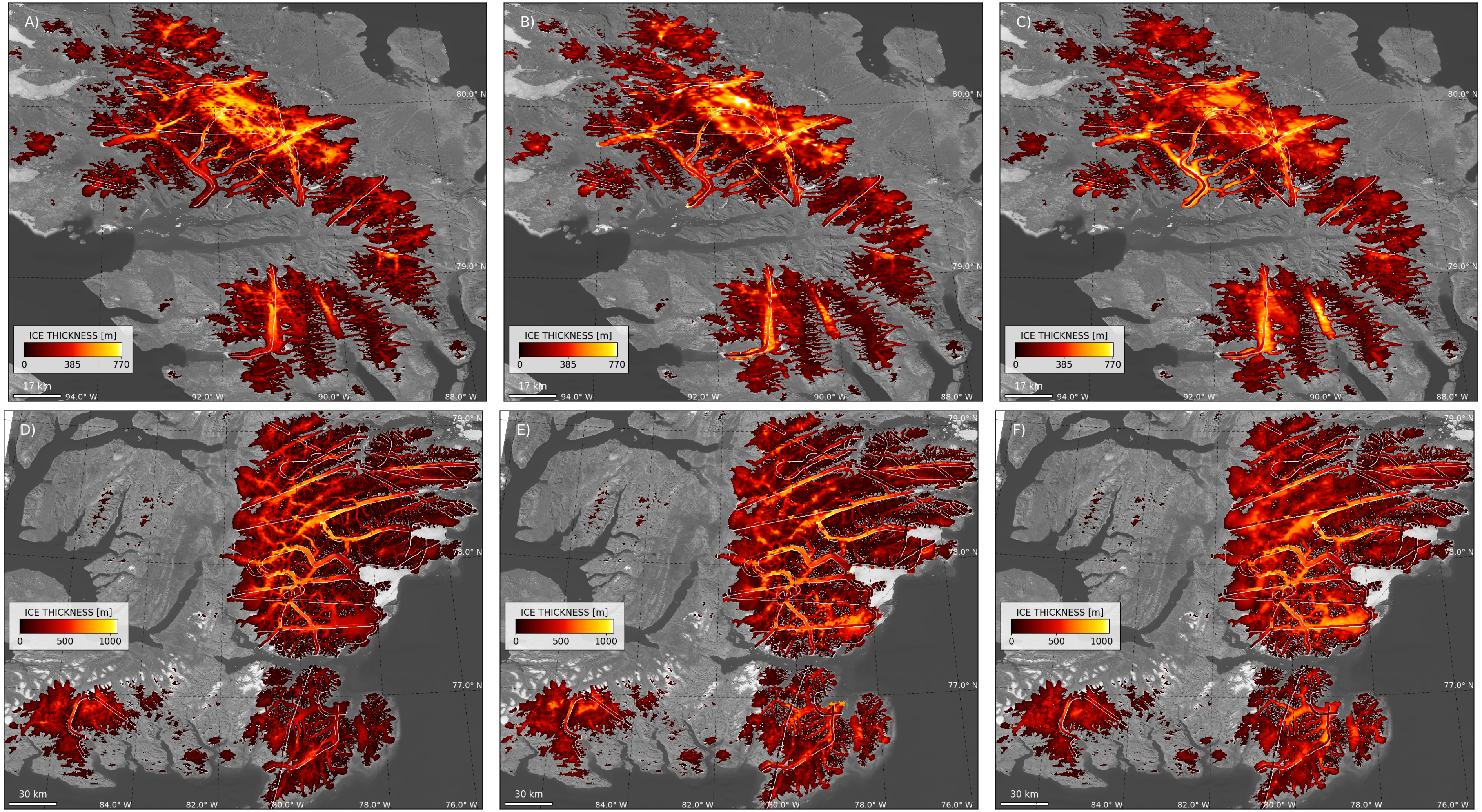

Figure S5: Baffin Island North (Canadian Arctic South, RGI 04). A=IceBoost v2.0 ; B=Millan et al. 2022 [2]; C=Farinotti et al. [3]. Zoom in for best view.

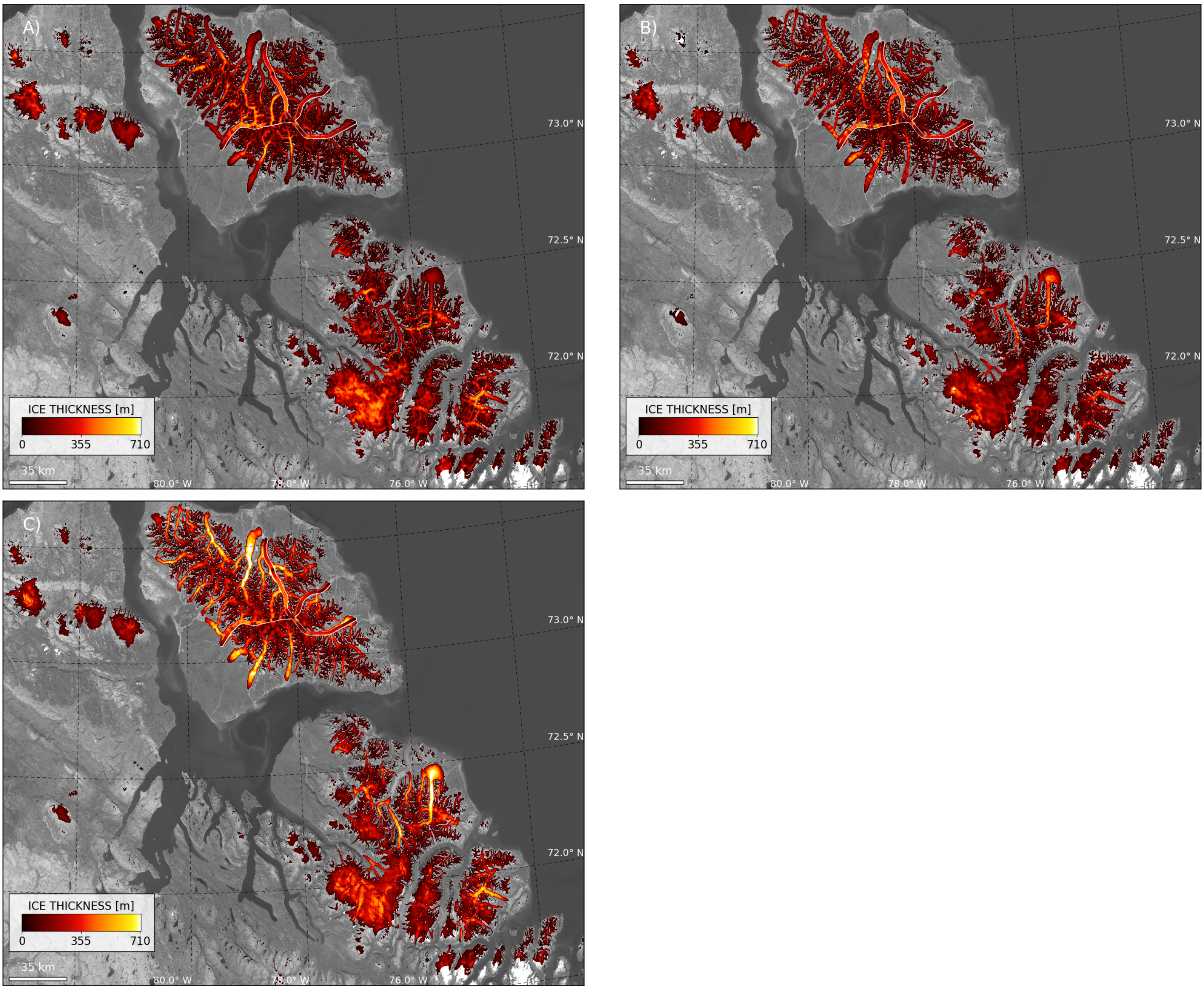

Figure S6: Canadian Arctic South (RGI 04). Top: Baffin Island Central. Bottom: Baffin Island South (Can. Arctic South). A,D=IceBoost v2.0; B,E=Millan et al. [2]; C,F=Farinotti et al. [3]. Zoom in for best view.

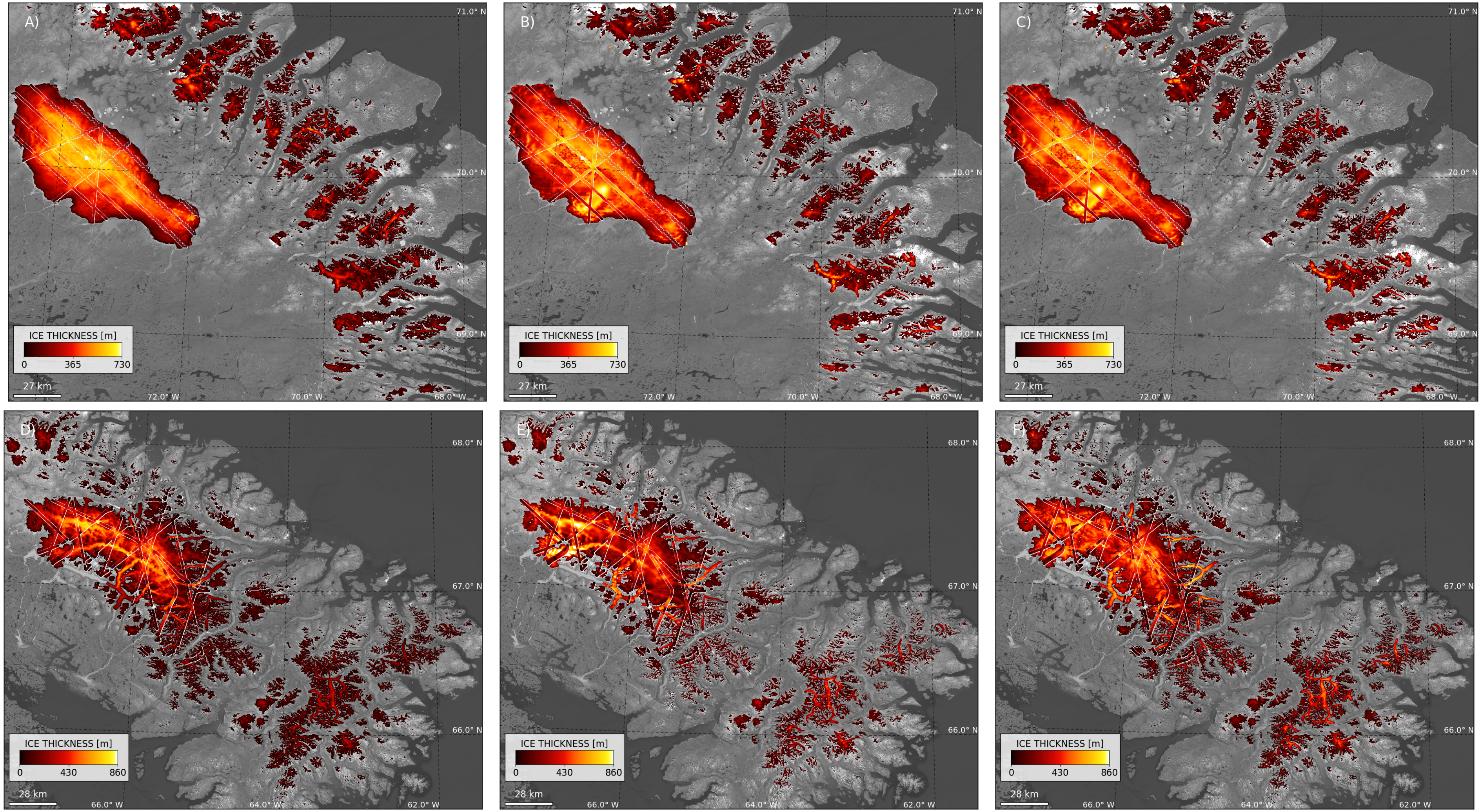

S5.3 Russian Arctic (RGI 09)

Table S7: Russian Arctic ice volumes estimated by different models. All units are $10^3\,\mathrm{km}^3$.

Russian Arctic (RGI 09)	IceBoost v2.0	Millan et al. [2]	Farinotti et al. [3]
Total	12.8 ± 3.0	15.5 ± 3.9	14.6 ± 3.8
- Novaya Zemlya	6.556 ± 1.307	7.595	7.080
- Severnaya Zemlya	4.287 ± 1.023	4.929	4.976
- Franz Josef Land	1.864 ± 0.6397	2.810	2.412
- Others	0.093 ± 0.030	0.166	0.132

No measurements from the Russian Arctic are available in our training dataset to validate any of the models, so only qualitative comparisons are possible (Figs. S7-S8). Farinotti's model exhibits strong discontinuities between neighboring basins. Millan's inversion appears realistic outside the low-sloping, fast-flowing termini of the Academy of Sciences Ice Cap, while suspiciously large ice thicknesses (> 600 meters) are estimated in these regions. IceBoost produces the shallowest ice across all models, and its regionally integrated ice volume is lower than the other two estimates. Data is needed everywhere in the Russian Arctic.

Figure S7: Franz Josef Land (Russian Arctic, RGI 09) modeled with IceBoost v2.0 (A), Millan et al. (B, [2]) and Farinotti et al. (C, [3]). Zoom in for best view.

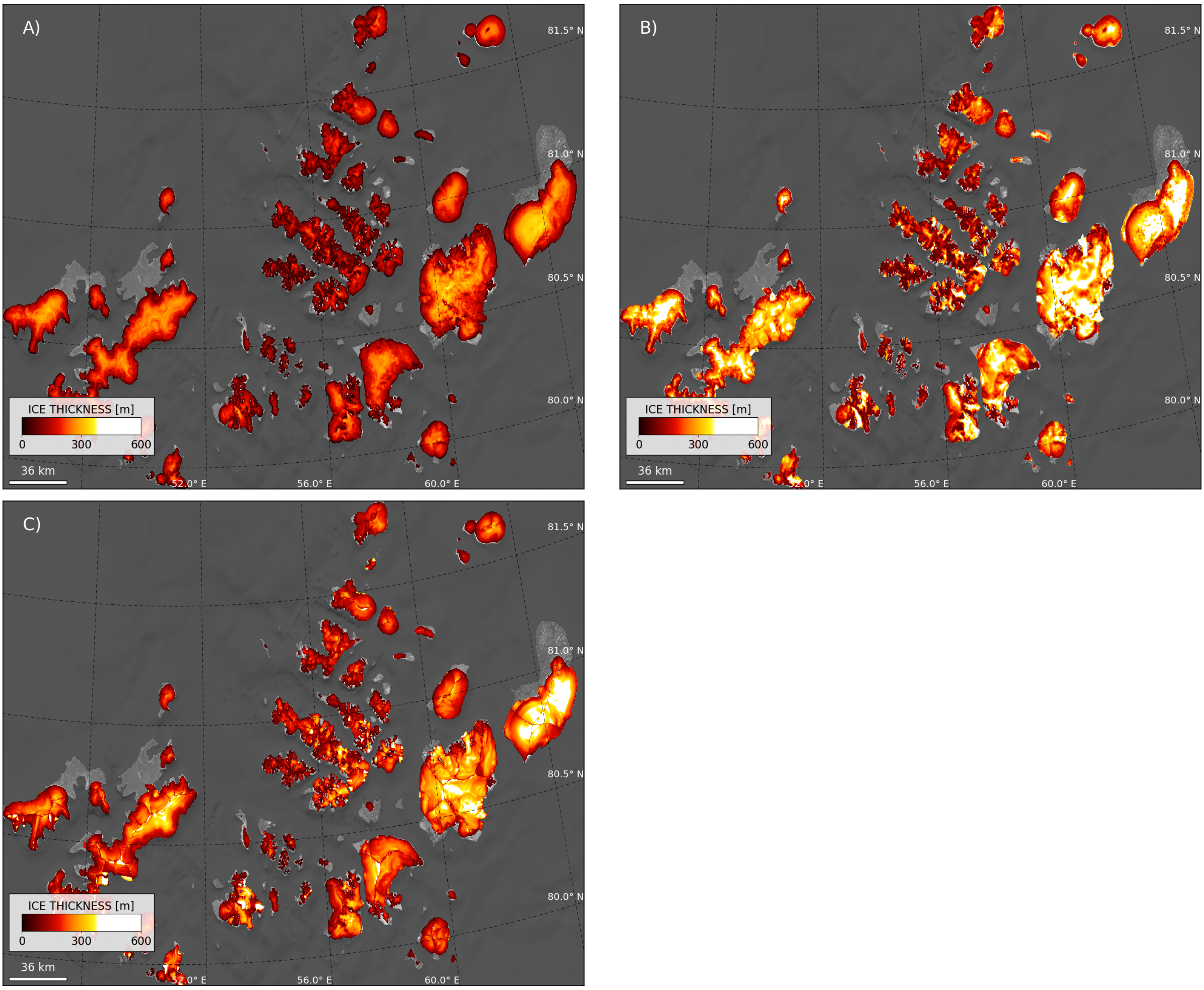

Figure S8: Russian Arctic (RGI 09). Top: Novaya Zemlya; Bottom: Severnaya Zemlya. A=IceBoost v2.0; B=Millan et al. [2]; C=Farinotti et al. [3]. Zoom in for best view.

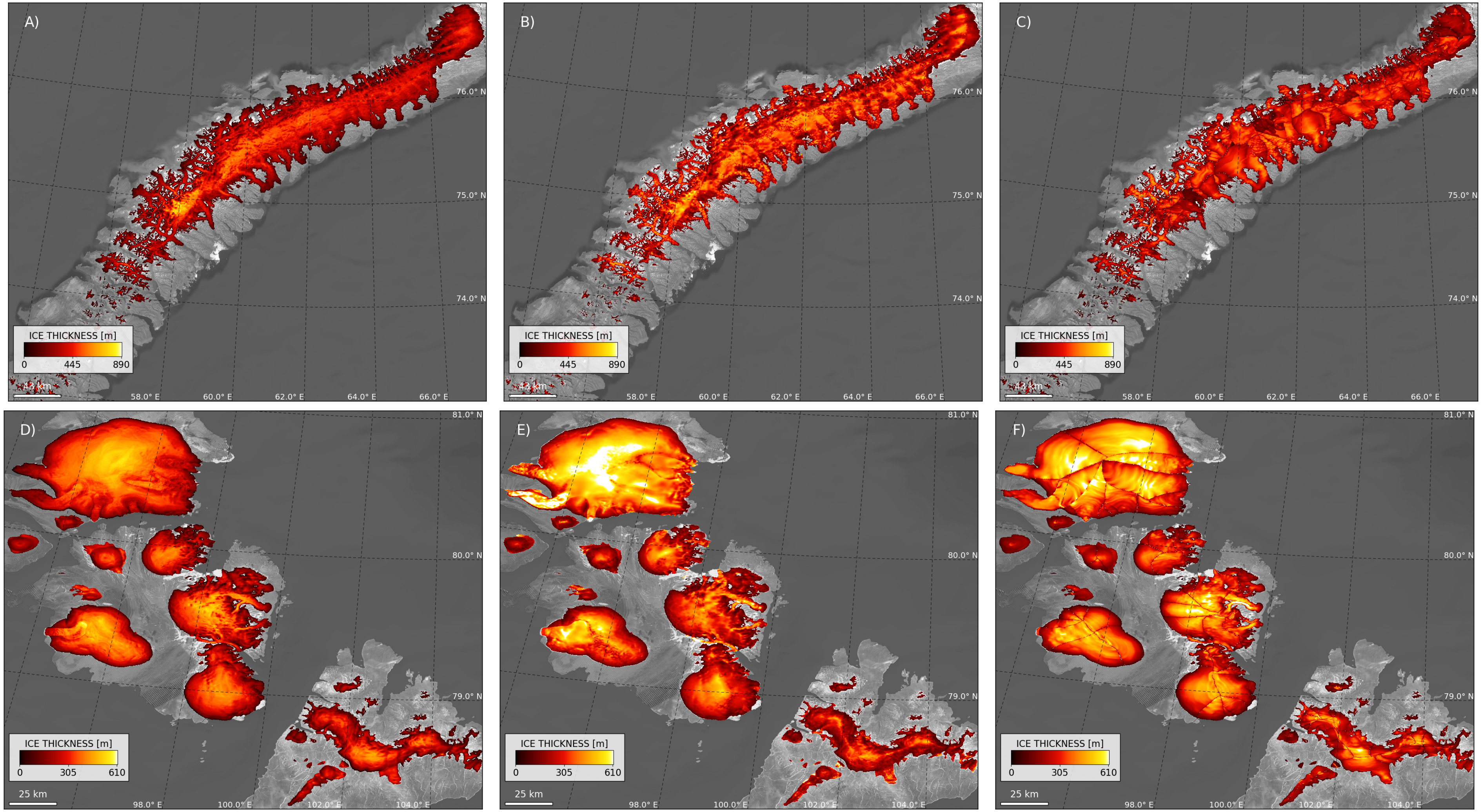

S5.4 Greenland periphery (RGI 05)

Table S8: Greenland periphery ice volumes estimated by different models. All units are 10^3 km^3.
* Central East Greenland includes 256 additional glaciers modeled with IceBoost, not included in [2, 3].

Greenland periphery (RGI 05)	IceBoost v2.0	Millan et al. [2]	Farinotti et al. [3]
Total	13.2 ± 4.6	11.8 ± 3.7	15.7 ± 4.1
- North Greenland and Hans Tausen Ice Cap	2.461 ± 1.009	1.734	2.556
- Flade Isblink	3.450 ± 1.014	3.689	4.844
- Sukkertoppen Ice Cap	1.17920 ± 0.3496	0.852	1.33788
- Central East Greenland*	2.611 ± 0.777*	1.882	2.182
- Others	3.4988 ± 1.45	3.643	4.78012
Glaciers with connection to the ice sheet	IceBoost v2.0	BedMachine v5 [4]	
- Geikie plateau	9.57061 ± 1. 953	5.100 ± 1.516	

The comparison between IceBoost and BedMachine v5 [4] over the Geikie Plateau (Fig. S9) suggests that the kriging and streamline-diffusion techniques used in BedMachine are unable to resolve many local features and ice streams in this region. IceBoost predicts nearly twice the total ice volume. We note that the forthcoming BedMachine v6 release will provide an updated thickness map for this area.

Ice thickness estimates differ depending on the modeling approach in the Northern Greenland periphery (Fig. S10). IceBoost and Millan's estimates diverge markedly over the Hans Tausen Ice Cap and the Freuchen Land peninsula: IceBoost predicts thicker ice and ∼40% more volume. No measurements exist to validate any of the models in this region.

In Central East Greenland (Scoresby Land), differences are also present (Fig. S10), though they are largely confined to the deepest portions of fjord glaciers and are on the order of 100–300 m. IceBoost shows the best agreement with radar profiles over the Renland Ice Cap.

Significant discrepancies also appear across Flade Isblink, Greenland's largest ice cap (Fig. S11). IceBoost agrees most closely with existing data, indicating that the ice cap is everywhere no more than ∼150 m above sea level. Millan and Farinotti both predict ice that is too thick. Smaller glaciers on the Kronprins Christian Land peninsula are consistent across models.

In western Greenland, over the Sukkertoppen Ice Cap, IceBoost estimates ice thicknesses of up to ∼500 meters in the northern sector and up to ∼1000 meters in the southern basin.

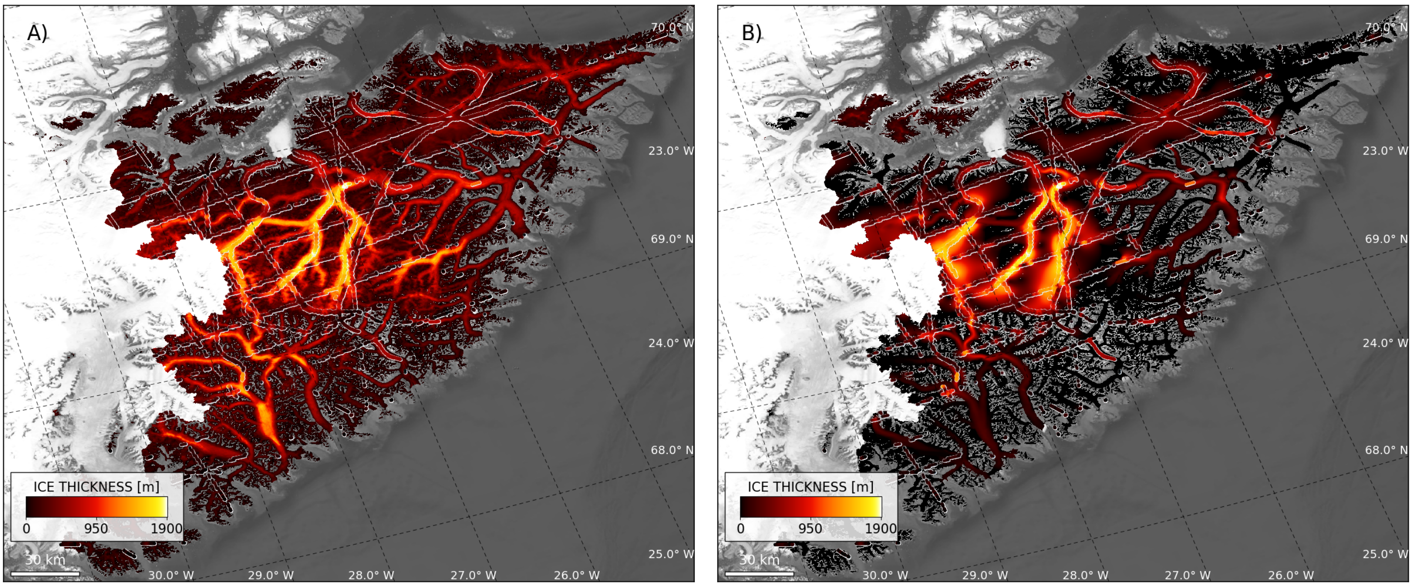

Figure S9: Geikie Plateau (East Greenland, RGI 05). A=IceBoost v2.0; B=BedMachine v5 [4]. Zoom in for best view.

Figure S10: Greenland Periphery (RGI 05). Top: Hans Tausen ice cap; Bottom: Central East Greenland. A,D=IceBoost v2.0; B,E=Millan et al. [2]; C,F=Farinotti et al. [3]. Central East Greenland includes 256 additional glacier polygons modeled with IceBoost. Zoom in for best view.

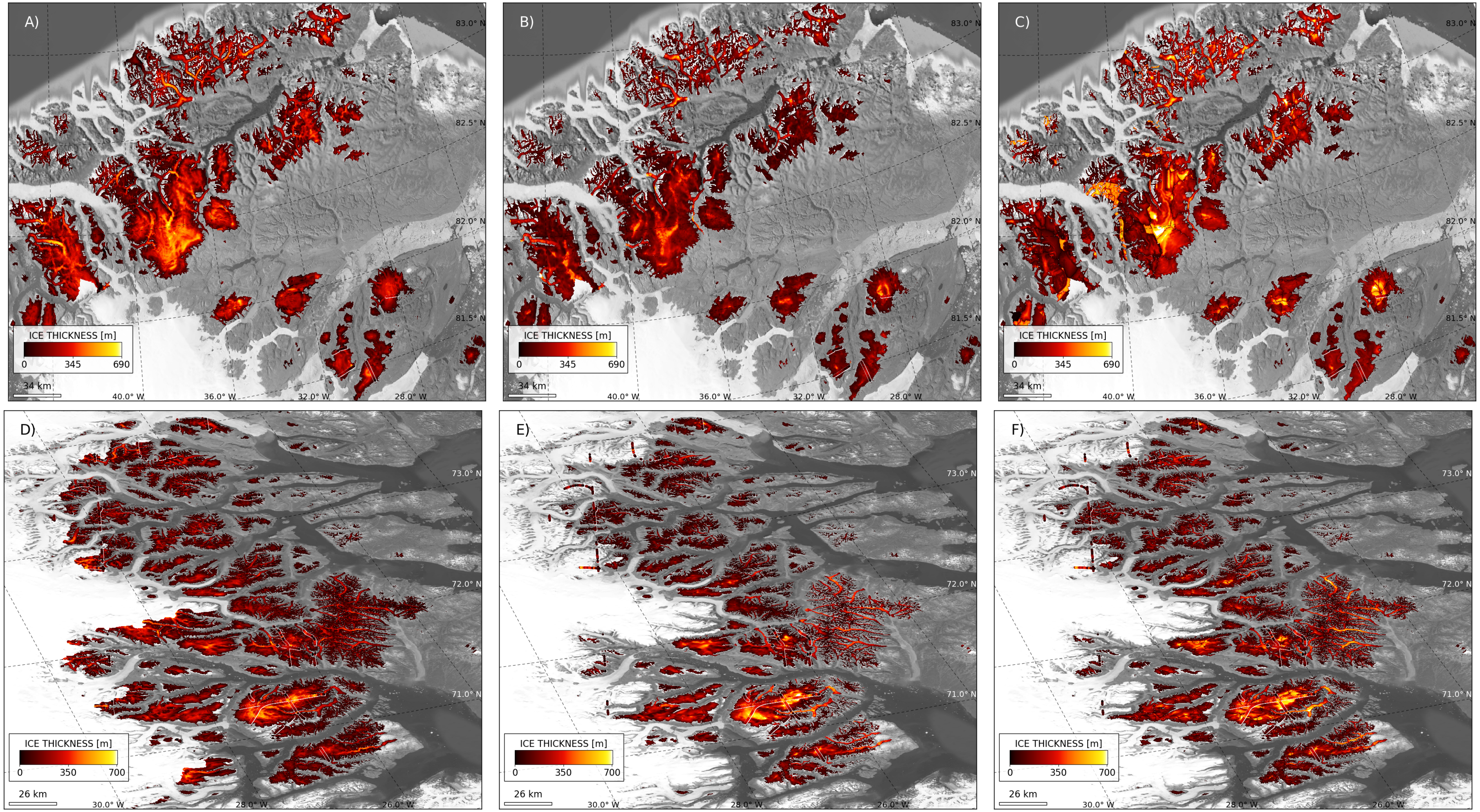

Figure S11: Greenland Periphery (RGI 05). Top: Flade Isblink; Bottom: Sukkertoppen ice cap. A,D=IceBoost v2.0; B,E=Millan et al. [2]; C,F=Farinotti et al. [3]. Zoom in for best view.

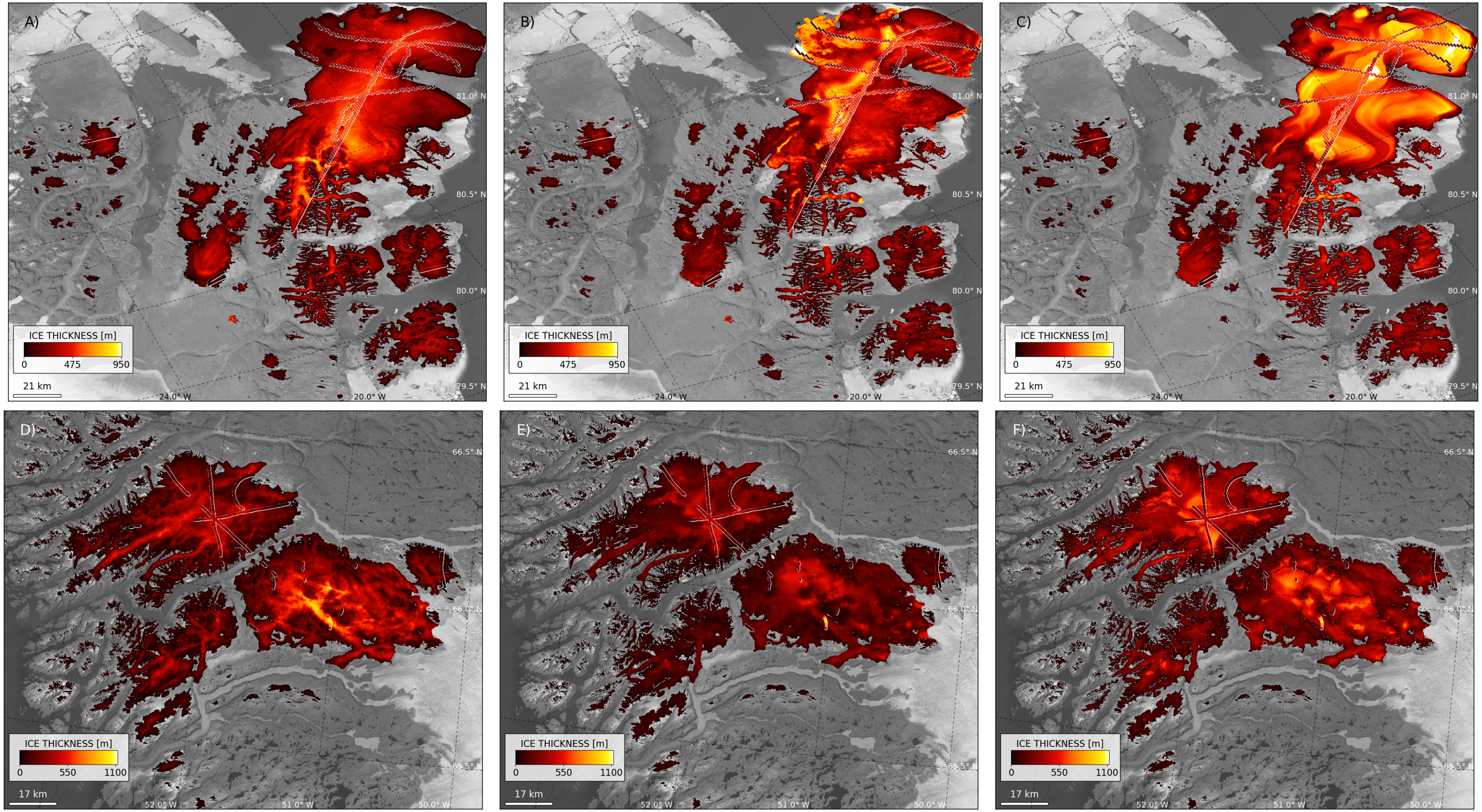

S5.5 Asia (RGI 13-14-15)

Table S9: Asia ice volumes estimated by different models. All units are 10^3 km^3.

Region	IceBoost v2.0	Millan et al. [2]	Farinotti et al. [3]
Total Central Asia (RGI 13)	3.8 ± 2.2	4.4 ± 2.7	3.27 ± 0.85
- Pamir	0.589 ± 0.275	0.671	0.497
- Tian Shan	0.463 ± 0.197	0.552	0.405
- Western Kulun Mountains	0.587 ± 0.267	0.624	0.388
- Others	2.161 ± 1.461	2.553	1.98
Total South Asia West (RGI 14)	3.8 ± 1.5	3.8 ± 2.4	2.87 ± 0.74
- Karakoram range	2.991 ± 1.164	2.710	2.062
- Others	0.809 ± 0.336	1.09	0.808
Total South Asia East (RGI 15)	1.0 ± 0.5	1.2 ± 0.8	0.88 ± 0.23
- Eastern Himalayas	0.233 ± 0.085	0.328	0.182
- Others	0.767 ± 0.415	0.872	0.698

Few-to-no measurements have been collected in Asia. Unlike other data-sparse Arctic regions (such as the Russian Arctic), the feature space in this region is likely unrepresented or outside the domain covered by the training data. As a result, the model operates in a highly generative regime. Elevations can be extreme, slopes very steep, velocity fields contain large gaps and significant outliers, and the mass-balance input may be a crude approximation. Yet, the IceBoost inversion appears realistic (Figs. S12, S13). The Karakoram stands out: IceBoost produces shallower thick-ice and thicker shallow-ice regions compared to both Millan and Farinotti (Fig. S13). However, given the absence of ground-truth data and the strongly out-of-distribution feature space, we cannot claim that the machine-learning approach yields an improvement over existing methods in this region.

Figure S12: Central Asia (RGI 13). Top: Tian Shan; Bottom: West Kulun mountains. A,D=IceBoost v2.0; B,E=Millan et al. [2]; C,F=Farinotti et al. [3]. Zoom in for best view.

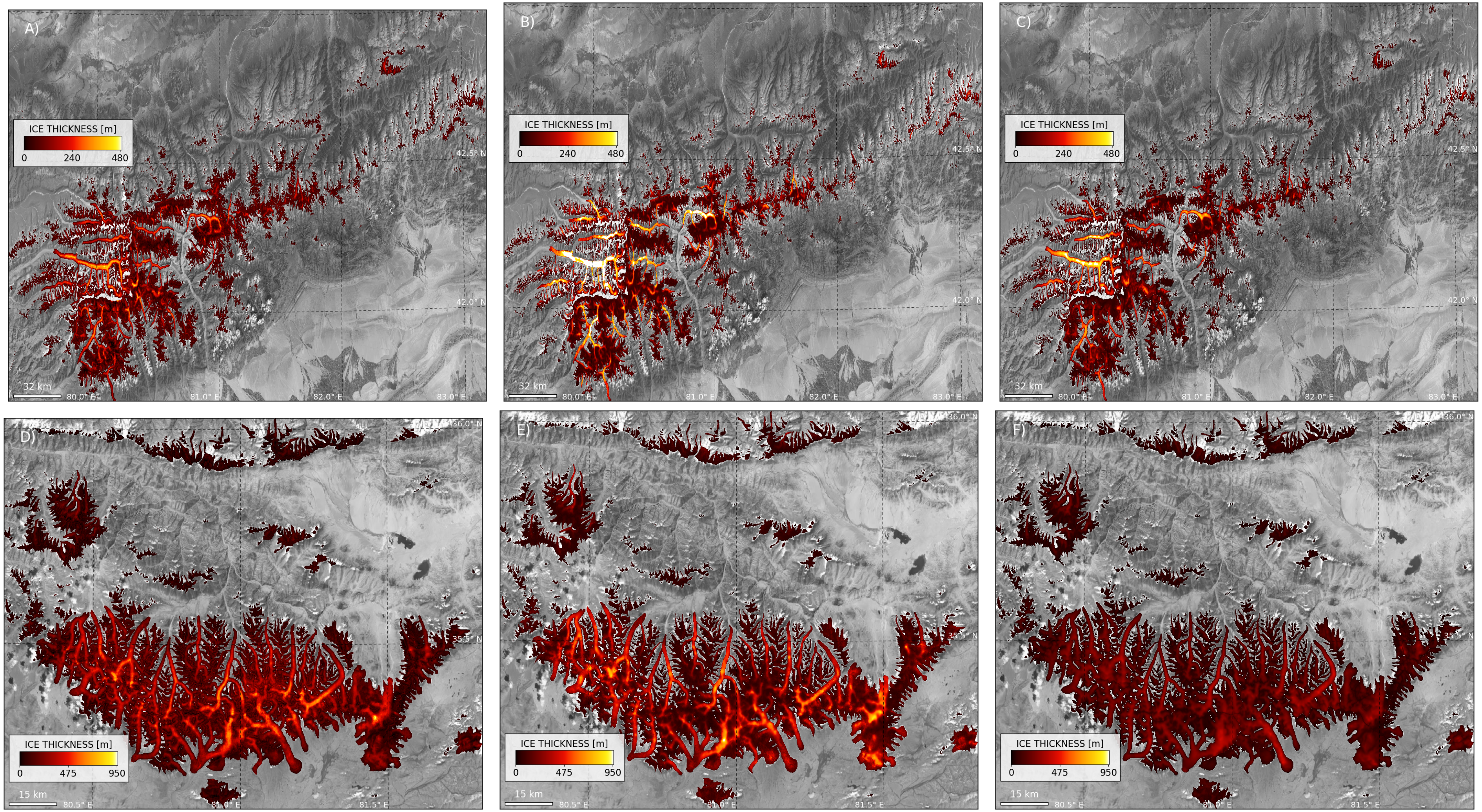

Figure S13: Top: Karakoram range (RGI 14); Bottom: Eastern Himalayas (RGI 15). A=IceBoost v2.0; B=Millan et al. [2]; C=Farinotti et al. [3]. Zoom in for best view.

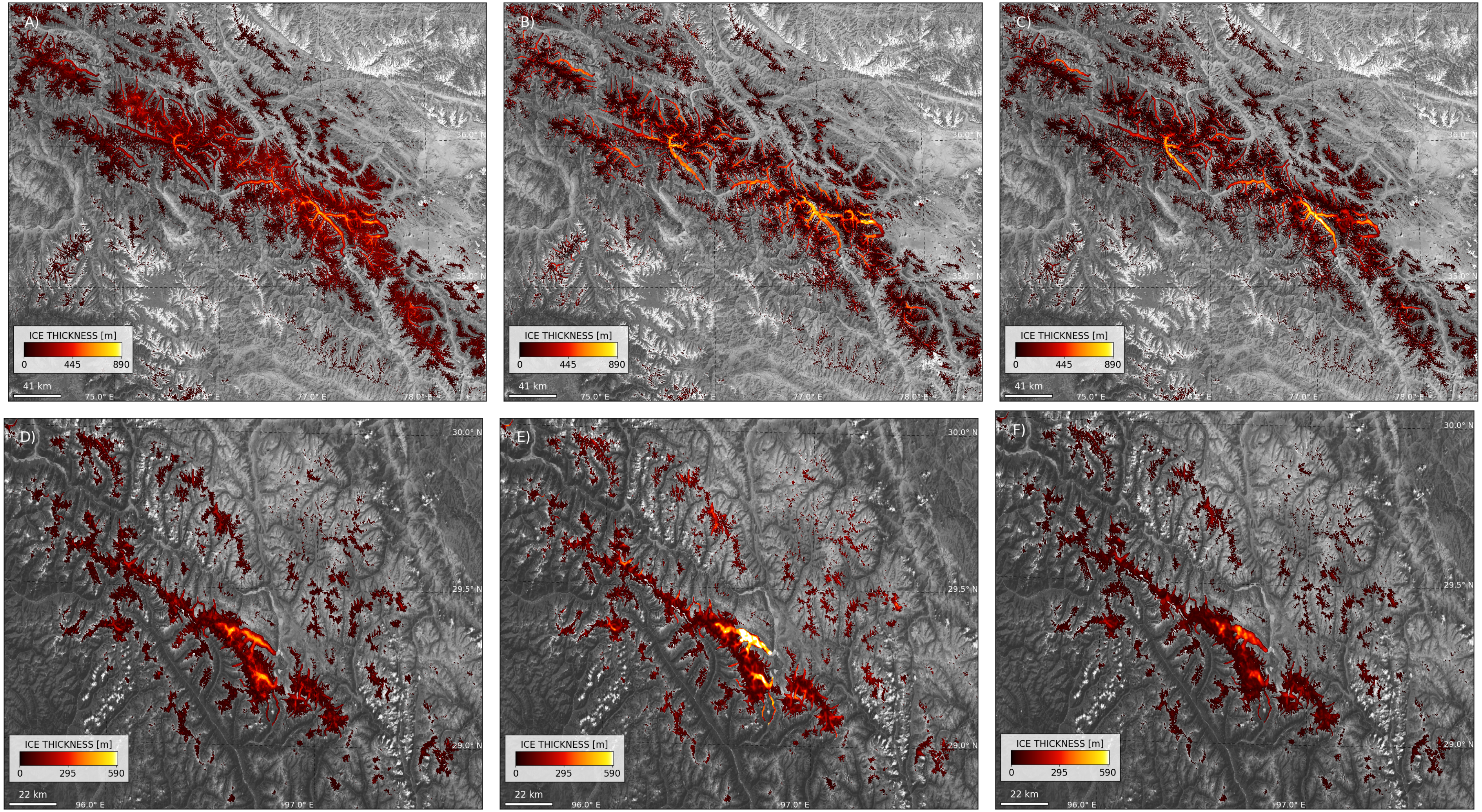

S5.6 Southern Andes (RGI 17)

Table S10: Southern Andes ice volumes estimated by different models. All units are 10^3 km^3.

Southern Andes (RGI 17)	IceBoost v2.0	Millan 2022 [2]	Farinotti 2019 [3]	Fürst 2024 [8]	Millan 2019 [9]
Total	6.8 ± 1.3	5.9 ± 1.6	5.3 ± 1.4	-	-
- Northern Patagonian Icefield	1.627 ± 0.272	1.224	1.105	1.150	1.147
- Southern Patagonian Icefield	4.287 ± 0.701	3.928	3.507	4.182	3.826
- Cordillera Darwin	0.357 ± 0.097	0.250	0.307	-	-
- Others	0.529±0.23	0.498	0.381	-	-

Over the Patagonian Icefields, we add to the set of model comparisons the inversions by Fürst et al. 2024 [8] and by Millan et al. 2019 [9]. The former uses a mass-conservation approach refined by a shallow ice approximation; the second uses a gravity-based inversion.

In the interior of the Northern Patagonian Icefield (NPI), IceBoost and the models by Fürst et al. (2024) [8] and Millan et al. (2019) [9] reproduce the high-thickness measurements (Fig. S14). However, IceBoost predicts thicker ice than the other models along the steep, mountainous terrain near ice-free nunataks. In the eastern sector of the icefield, none of the models can reproduce the thick-ice measurements included in the training dataset. These values may be unrealistically high and could bias IceBoost toward predicting excessively thick ice.
Overall, both the data and three of the five models indicate that the central portion of the icefield is likely about 1000–1200 m thick. The shallower, more rugged areas are more uncertain and show larger discrepancies across estimates. IceBoost may be positively biased, and its bootstrap performance analysis (Supp. Info. Fig. S1) supports this possibility. Its error in the Southern Andes is the highest among all global regions, suggesting that some training data there may be unreliable.
In the Northern Patagonian Icefield, we are not more confident in IceBoost than in the other models. Suspect thick-ice measurements in the eastern NPI make the reliability of IceBoost in this region uncertain, although it may still perform well over the thick central region and outlet glaciers. The San Quintín Glacier and Steffen Glacier are modeled to be grounded below sea level. The model is supported by data.

Reconstructions of the Southern Patagonian Icefield (SPI) using IceBoost, the method by Fürst et al. (2024) and the shallow-ice approximation [2] are broadly similar (Fig. S15). In contrast, Farinotti's ensemble appears generally too shallow. The gravity inversion by Millan et al. (2019) [9] shows very shallow ice over steep mountainous terrain and very thick ice elsewhere. We find that the termini of the Pío XI Glacier and Occidental Glacier are grounded below sea level, by up to roughly 500 m. This result cannot be confirmed by measurements, as these areas remain unsurveyed. Both IceBoost and observations indicate that the termini of the George Montt Glacier, Bernardo Glacier, Upsala Glacier and Tyndall Glacier are also grounded below sea level.
Across both Patagonian Icefields, available reconstructions still diverge substantially. Disagreement occurs in both thick- and thin-ice regions. We argue that measurements would be beneficial over thin ice over the NPI, and everywhere over the SPI.

Figure S14: Northern Patagonian Icefield (RGI 17). A=IceBoost v2.0; B=Millan et al. 2022 [2]; C=Farinotti et al. [3]; D=Fürst et al. [8]; E=Millan et al. 2019 [9]. Zoom in for best view.

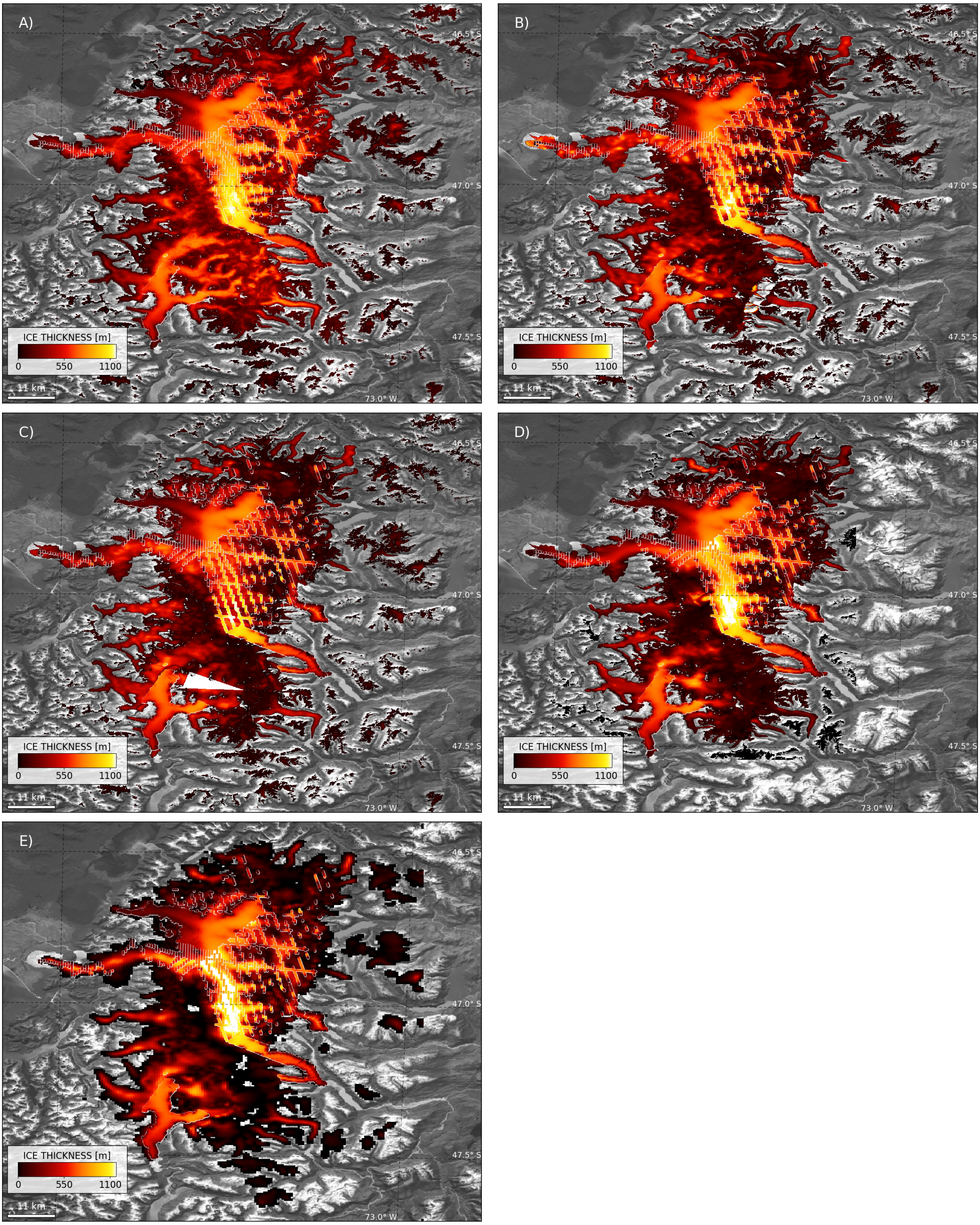

Figure S15: Southern Patagonian Icefield (RGI 17). A=IceBoost v2.0; B=Millan et al. 2022 [2]; C=Farinotti et al. [3]; D=Fürst et al. [8]; E=Millan et al. 2019 [9]. Zoom in for best view.

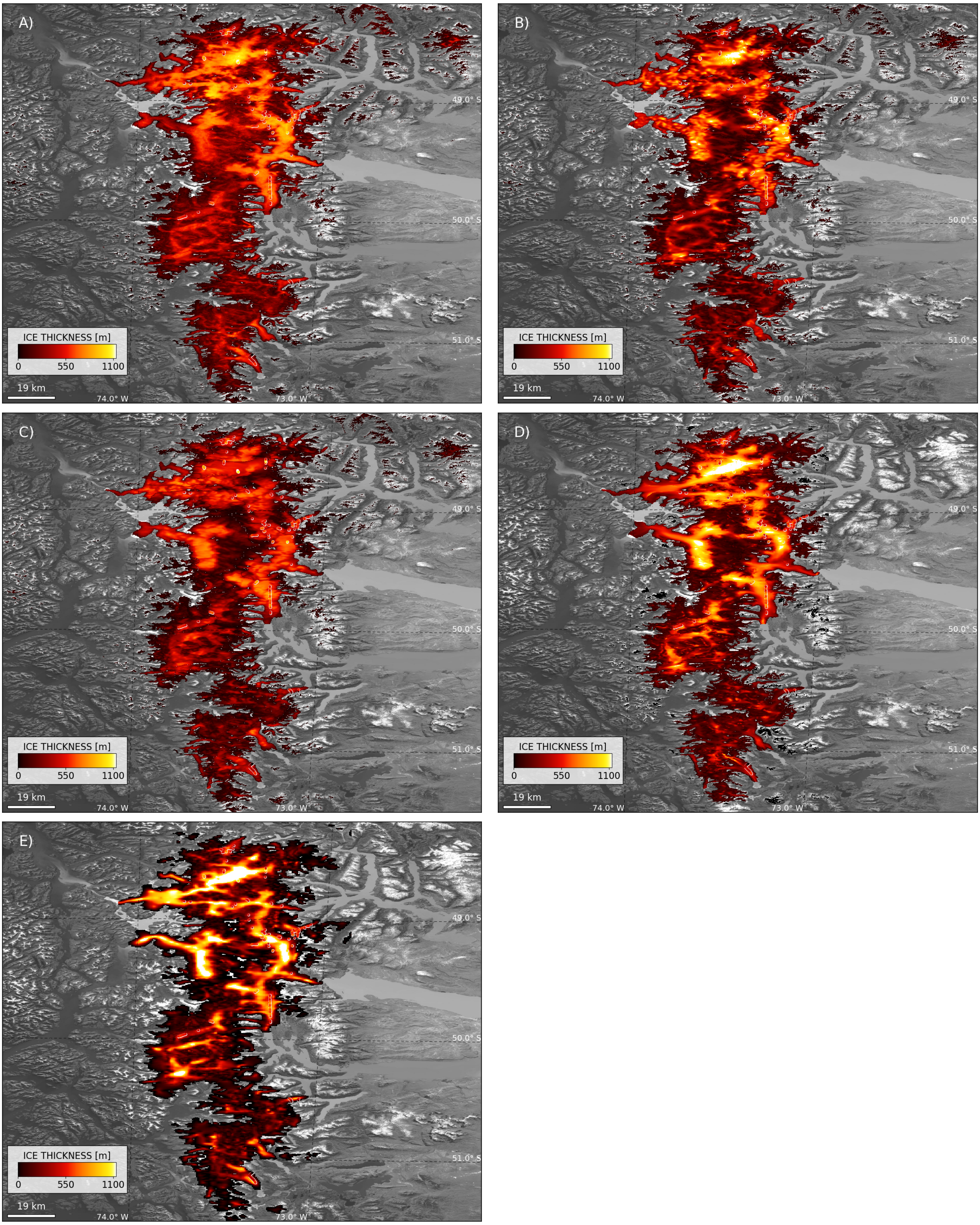

References

[1] N. Maffezzoli, E. Rignot, C. Barbante, T. Petersen, and S. Vascon. A gradient-boosted tree framework to model the ice thickness of the world's glaciers (iceboost v1.1). *Geoscientific Model Development*, 18(9):2545–2568, 2025.

[2] Romain Millan, Jérémie Mouginot, Antoine Rabatel, and Mathieu Morlighem. Ice velocity and thickness of the world's glaciers. *Nature Geoscience*, 15(2):124–129, 2022.

[3] Daniel Farinotti, Matthias Huss, Johannes J Fürst, Johannes Landmann, Horst Machguth, Fabien Maussion, and Ankur Pandit. A consensus estimate for the ice thickness distribution of all glaciers on earth. *Nature Geoscience*, 12(3):168–173, 2019.

[4] Mathieu Morlighem and Et Al. Icebridge bedmachine greenland, version 5, 2022.

[5] Mathieu Morlighem. Measures bedmachine antarctica, version 3, 2022.

[6] BS Tober, JW Holt, MS Christoffersen, M Truffer, CF Larsen, DJ Brinkerhoff, and SA Mooneyham. Comprehensive radar mapping of malaspina glacier (sít'tlein), alaska—the world's largest piedmont glacier—reveals potential for instability. *Journal of Geophysical Research: Earth Surface*, 128(3):e2022JF006898, 2023.

[7] Abigail Dalton, Wesley Van Wychen, Luke Copland, Laurence Gray, and David Burgess. Seasonal and multiyear flow variability on the prince of wales icefield, ellesmere island: 2009–2019. *Journal of Geophysical Research: Earth Surface*, 127(4):e2021JF006501, 2022.

[8] Johannes J Fürst, David Farías-Barahona, Norbert Blindow, Gino Casassa, Guisella Gacitúa, Michèle Koppes, Emanuele Lodolo, Romain Millan, Masahiro Minowa, Jérémie Mouginot, et al. The foundations of the patagonian icefields. *Communications Earth & Environment*, 5(1):142, 2024.

[9] Romain Millan, Eric Rignot, A Rivera, Vincent Martineau, Jérémie Mouginot, Rodrigo Zamora, Jose Uribe, G Lenzano, Basile De Fleurian, Xiaojian Li, et al. Ice thickness and bed elevation of the northern and southern patagonian icefields. *Geophysical Research Letters*, 46(12):6626–6635, 2019.
